# Fate of a Naive T Cell: A Stochastic Journey

**DOI:** 10.3389/fimmu.2019.00194

**Published:** 2019-03-06

**Authors:** Luis de la Higuera, Martín López-García, Mario Castro, Niloufar Abourashchi, Grant Lythe, Carmen Molina-París

**Affiliations:** ^1^Department of Applied Mathematics, School of Mathematics, University of Leeds, Leeds, United Kingdom; ^2^Grupo Interdisciplinar de Sistemas Complejos and DNL, Universidad Pontificia Comillas, Madrid, Spain; ^3^Department of Statistical Science, University College London, London, United Kingdom

**Keywords:** T cell, stochastic model, continuous-time Markov chain, single cell, cellular fate, migration, division, apoptosis

## Abstract

The homeostasis of T cell populations depends on migration, division and death of individual cells (1). T cells migrate between spatial compartments (spleen, lymph nodes, lung, liver, etc.), where they may divide or differentiate, and eventually die (2). The kinetics of recirculation influences the speed at which local infections are detected and controlled (3). New experimental techniques have been developed to measure the lifespan of cells, and their migration dynamics; for example, fluorescence-activated cell sorting (4), *in vitro* time-lapse microscopy (5), or *in vivo* stable isotope labeling (e.g., deuterium) (6). When combined with mathematical and computational models, they allow estimation of rates of migration, division, differentiation and death (6, 7). In this work, we develop a stochastic model of a single cell migrating between spatial compartments, dividing and eventually dying. We calculate the number of division events during a T cell's journey, its lifespan, the probability of dying in each compartment and the number of progeny cells. A fast-migration approximation allows us to compute these quantities when migration rates are larger than division and death rates. Making use of published rates: (i) we analyse how perturbations in a given spatial compartment impact the dynamics of a T cell, (ii) we study the accuracy of the fast-migration approximation, and (iii) we quantify the role played by direct migration (not via the blood) between some compartments.

## 1. Introduction

T cells are descendants of bone marrow progenitors that migrated to the thymus and underwent processes of maturation, gene rearrangement and selection (8). The surface of a T cell is populated with tens of thousands of copies of a T-cell receptor. A repertoire of T cells is maintained in a mammal's body that enables recognition of and response to the many benign and pathogenic microorganisms that are encountered over its lifetime, although the T-cell receptor of any individual cell only recognizes a tiny fraction of them (9, 10). An individual T cell may circulate between different tissues of the body for months or years, never encountering cognate antigen. Their interactions with self antigens, generally weak, are occasionally strong enough to cause one round of cell division. Strong interaction between the T-cell receptor and non-self antigens, mounted on MHC on the surface of antigen-presenting cells in lymph nodes (11), initiates a programme of multiple rounds of cell division and phenotypic changes that generate effector and memory T cells with different lifetimes and migration patterns (2, 12–15).

Blood is a dynamic conduit through which T cells pass, in homeostasis and during immune responses (16). Blood is also the only tissue from which it is easy to obtain samples of T cells from healthy humans, although only about two percent of the body's T cells are in the blood at any one time (17, 18). The fraction of T cells found in a particular tissue depends on how likely a T cell is to enter the tissue and on how long it stays there. At any one time, for example, the fraction of T cells in lymph nodes and spleen is large, not because a T cell in the blood is most likely to go there, but because, when they do enter, they remain there a long time (3). Direct counts of T cell numbers in organs of mice are sometimes possible (19, 20); direct measurement of the kinetics of recirculation is more difficult. Mathematical models of the full kinetics of recirculation are the basis of a systematic extrapolation from measurements to residence times and migration probabilities.

Ganusov and Auerbach (3) constructed a model, based on experimental data, in which the migration history of a T cell consists of short intervals in the blood (less than a minute each) between longer sojourns in lung, liver, spleen and lymph nodes. We adopt their star-shaped migration topology pattern here. We also adopt a Markov description, in which the next event in the lifetime of a T cell (migration, division or death) is stochastic, but governed by parameters that depend only on the cell's current position. We treat a division event as the birth of one new cell, that follows the same rules as its mother, and a continuation of the life of another. In our modeling, we have in mind the homeostasis of naive CD4^+^ T cells, without explicitly taking the effect of aging (15, 19, 21) into account.

Novel labeling techniques are providing an increasing amount of information about recirculation and other properties at the single cell level (4–6), which lead to new hypotheses and new experiments aimed at elucidating the kinetic properties of a cell's journey. Techniques such as staining or barcoding are ideal for quantifying dynamics at the single cell level, since they are able to track individual cells, their interactions with the extra-cellular environment and other cells and to help understand single cell lifetime dynamics (22, 23).

Although they are able to provide a substantial amount of quantitative data, experimental techniques are still far from being able to provide a full picture of lymphocyte dynamics *in vivo*, even in mice (24, 25). Thus, a partnership between experimental and *in silico* approaches is required. Deterministic continuous time models (based on ordinary differential equations) are the usual approach to study the kinetics of cell recirculation (7, 26, 27) when describing large cell populations. On the other hand, these deterministic approaches can miss some crucial behavior due to the stochastic nature of cellular heterogeneity and cellular interactions (28, 29). Stochastic processes are more appropriate when studying observables at the single cell level, instead of at the population level (30, 31).

This work is inspired by these new experimental techniques, and by the work of Ganusov and Auerbach (3), where the authors analyse the kinetics of lymphocyte recirculation. Our aim is to show how new analytical approaches can be applied to these systems to study the stochastic journey of a single cell during its lifetime. Based on the assumption that there are many more migration events than division and death events, we propose a *fast-migration approximation*. Finally, we carry out a range of numerical experiments to test the approximation, and to show the impact that cellular events occurring in a given spatial compartment can have on the whole system.

## 2. Theory

### 2.1. Description of the General Model

**Figure 1:**
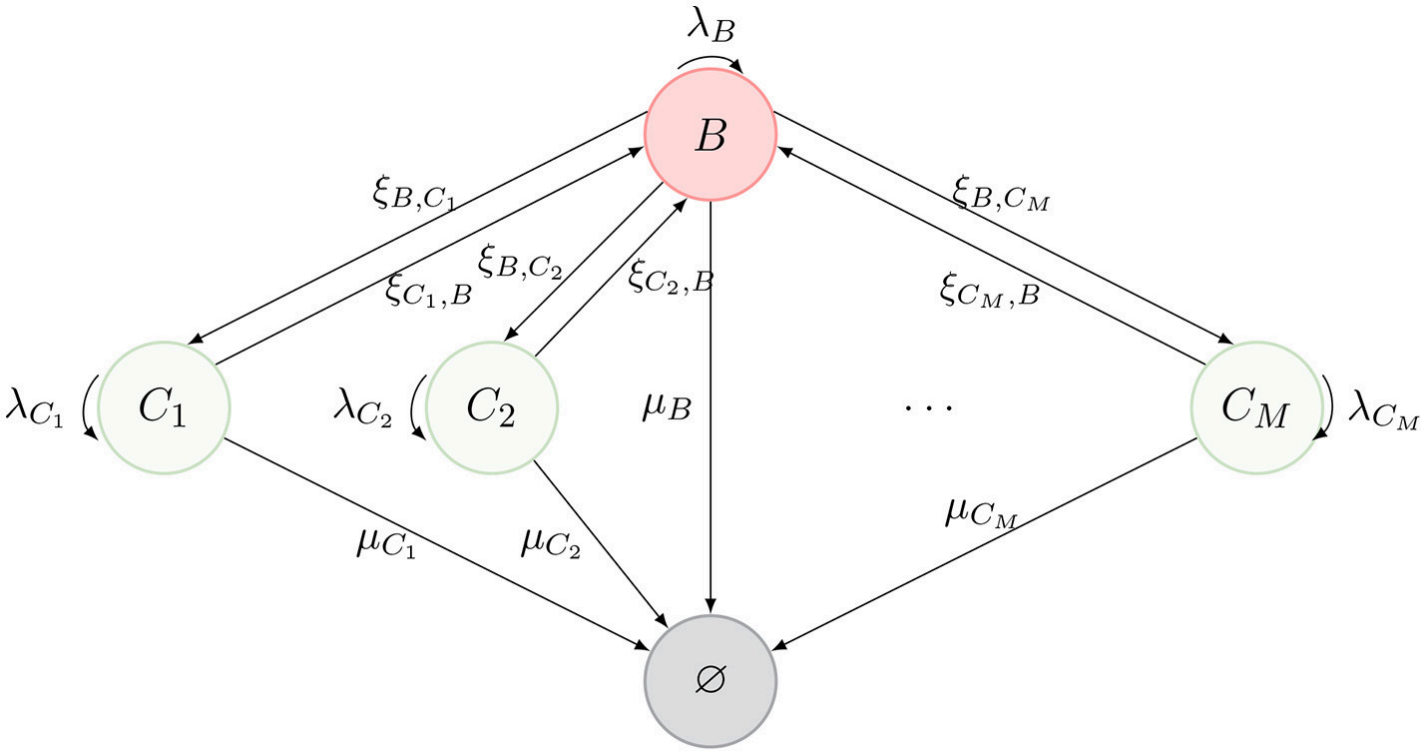
Schematic description of the model. The nodes represent spatial *compartments* where the CD4^+^ T cell can be located at a given time. The arrows connecting them represent the migration of the cell between compartments, with migration rates {(ξ_*B*,*C*_*j*__, ξ_*C*_*j*_, *B*_), *j* ∈ {1, …, *M*}}. Division events are represented by curved arrows with rates λ_*B*_ and {λ_*C*_*j*__, *j* ∈ {1, …, *M*}}. Finally, the state ∅ represents the death of the cell (with different death rates μ_*B*_ and {μ_*C*_*j*__, *j* ∈ {1, …, *M*}}, depending on the location where cell death takes place).

**Figure 2:**
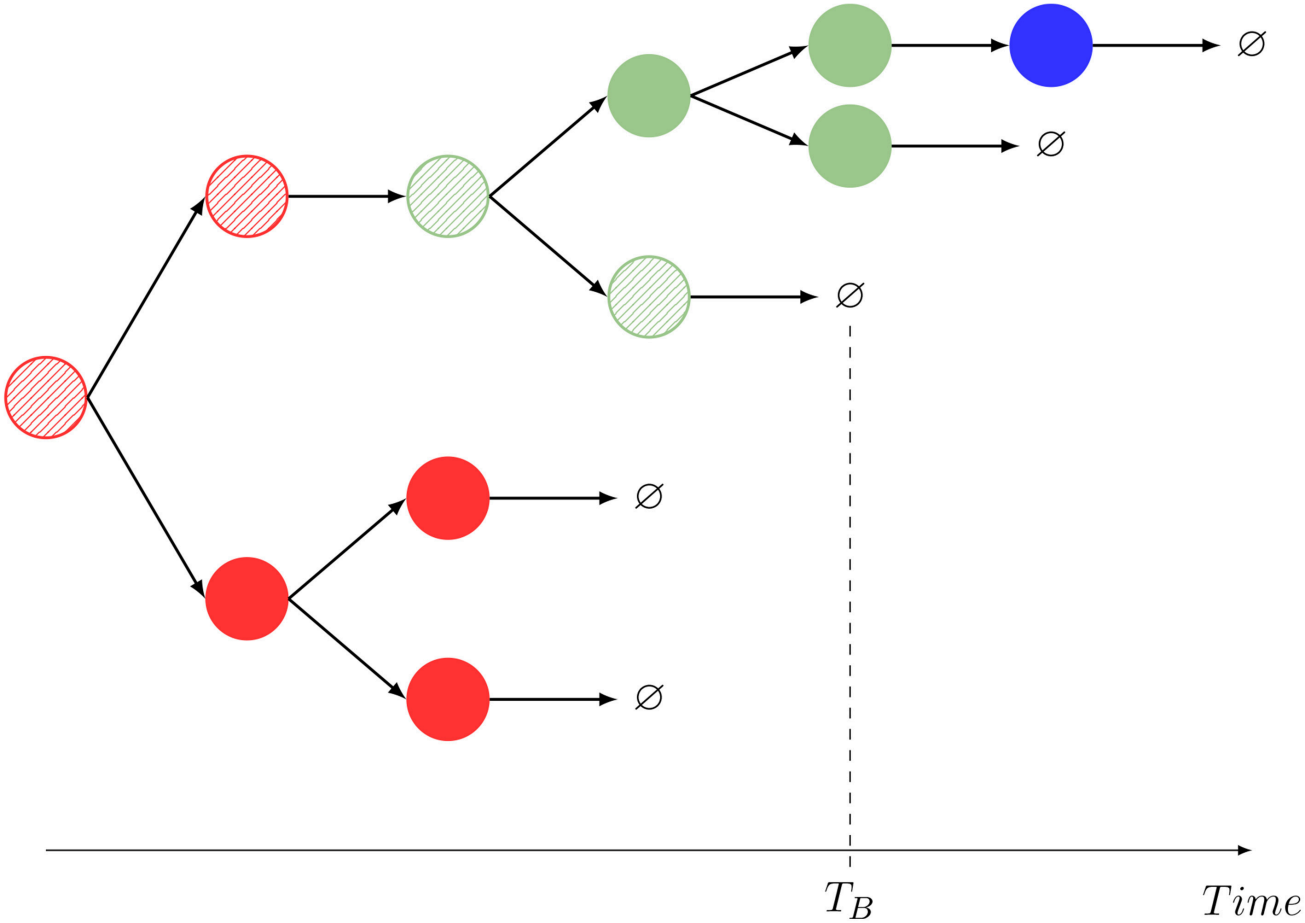
One realization of the stochastic process shown in Figure 1, and interpretation of the summary statistics. The dynamics mimics that of Hawkins et al. (35, Supplementary Material, Figure 2A) and Piltti et al. (36, Figure 2). In this example, *M* = 2, and a striped color identifies the original cell being tracked. A given color indicates the spatial location of each cell (red: blood, blue: *C*_1_, green: *C*_2_). In this realization, *G*_*B*_ = 11, *N*_*B*_ = *N*_*B*_(*B*) + *N*_*B*_(*C*_1_) + *N*_*B*_(*C*_2_) = 1 + 0 + 1 = 2. The compartment before death is *C*_2_.

We consider a model of a T cell that migrates between different spatial compartments, where it may divide one or more times, before ultimately dying. Inspired by the representation of Ganusov and Auerbach (3, Figure 2), these compartments can represent blood, lymph nodes, lung, liver, spleen and Peyer's patches. We denote the blood compartment by *B* and denote *M* additional compartments by {*C*_1_, …, *C*_*M*_} (see Figure 1).

The journey of a T cell during its lifetime is summarized by the diagram in Figure 1. A cell can migrate between compartments, divide or die (reaching the state ∅). Our model is an absorbing continuous-time Markov chain (CTMC) Y={Y(t): t≥0} defined on the space of states $S=\left\{B,C_{1},\ldots ,C_{M},\emptyset \right\}$, where *Y*(*t*) identifies the position of the cell at time *t* ≥ 0. We note that division does not affect the position of the cell, *Y*(*t*), and therefore, we keep division events in our description as events that leave the process in the same state, as described in Figure 1. When tracking a given T cell, if a division event occurs, one of the two resulting cells is the daughter, while the other is taken to be the original cell.

Our aims are: (i) to show how the dynamics of a T cell (see Figure 1) can be studied by means of a number of summary statistics (or *stochastic descriptors*) in section 2.2, inspired by current single cell experimental techniques; (ii) to present in section 2.3 a fast-migration approximation which allows us to simplify the analysis when migration rates are much larger than division and death rates; and (iii) to quantify the impact of changes occurring in a single spatial compartment (section 3).

### 2.2. Single Cell Descriptors

Recent studies have highlighted the importance of improving the existing experimental and analytic toolset for continuous single cell dynamics. While some tools such as TimeLapseAnalyzer (32) or TLM-Tracker (33) are fully automated, successful *in vitro* single cell tracking by long-term time-lapse microscopy usually requires combined automated methods and manual curation. It is worth mentioning here the recently developed single cell tracking and quantification software toolset consisting of The Tracking Tool and qTFy (34), which allows for robust and efficient analysis of large amounts of time-lapse imaging data, is not limited to specific cell types, and allows for some degree of manual curation after automated processing.

These and similar tools have led to the quantification of cellular dynamics corresponding to a single cell or the whole lineage descended from a *founder* cell. When this cellular dynamics is represented in terms of a stochastic process consisting of division, migration and death events, such as the one in Figure 1, our aim is to define and analyse a number of summary statistics that can be compared to the dynamics observed experimentally, at least in *in vitro* experiments. In particular, the Markovian representation of the process in Figure 1 allows us to make use of first-step arguments to analyse a number of summary statistics for the cellular dynamics. In this section, we present the summary statistics of interest together with exact formulæ for their computation, while the mathematical details to obtain these expressions can be found in the Appendix.

These summary statistics are directly inspired by data obtained from the experimental analysis of single cell dynamics and cell *pedigrees*. For example, when analysing a single founder B cell in *in vitro* experiments, Hawkins et al. (35) were able to obtain data regarding its lineage tree and quantified the times for cell division and death of the founder and descendent cells [see Figure 2A in Hawkins et al. (35, Supplementary Material)]. Similar dynamics and analysis can be found in Piltti et al. (36, Figure 2) for *in vitro* experiments with neural stem cells. On the other hand, if one was to consider a simulation of the stochastic process described in Figure 1, a realization would resemble Figure 2. In the same manner, in Reinhardt et al. (37), the authors show how the time-course of OT-II counts can be tracked in different locations *in vivo* (blood, spleen, lymph nodes, …). This experimental setup contains valuable information about total counts or even cumulative numbers in each spatial compartment. For long enough times, these counts could be directly linked to the total number of divisions in each compartment. This kind of long-time experiments can be found, for instance, in Masopust et al. (38) where CD8^+^ T cells were tracked for almost three months, or in Sathaliyawala et al. (39) where the count is made in humans at the time of death of the donors.

Motivated by these experimental achievements, we introduce different stochastic descriptors (also known as summary statistics). Not all of them can be straightforwardly quantified but, interestingly, combined they give information about specific aspects of the cellular dynamics that are unattainable using standard population dynamics approaches. In particular, the process in Figure 2, similarly to that in Hawkins et al. (35, Supplementary Material, Figure 2A) or Piltti et al. (36, Figure 2), can be quantified in terms of the following statistics:

- Lifetime of a CD4^+^ T cell and number of division events of this cell during its lifetime,
  $$\begin{array}{l} T_{i}=“lifetime\;of\;a\;given\;cell\;starting\;in\;compartment\;i”\;\\ \;=\;inf\left\{t\geq 0:\;Y\left(t\right)=\emptyset \;|\;Y\left(0\right)=i\right\},\\ N_{i}=“for\;a\;given\;cell\;starting\;in\;compartment\;i,\\ \;number\;of\;division\;events\;until\;it\;dies^{\prime \prime }, \end{array}$$
  for *i* ∈ {*B, C*_1_, …, *C*_*M*_}. If we define *m*_*i*_ = IE(*T*_*i*_) and ${\hat{m}}_{i}=\text{𝔼}\left(N_{i}\right)$, one can show that
  $$\begin{array}{l} m_{B}={\left(\mu_{B}+\overset{M}{\underset{i=1}{\sum }}\xi_{B,C_{i}}-\overset{M}{\underset{i=1}{\sum }}\xi_{B,C_{i}}\xi_{C_{i},B}{\left(\mu_{C_{i}}+\xi_{C_{i},B}\right)}^{-1}\right)}^{-1}\\ \;\times \left(\overset{M}{\underset{i=1}{\sum }}\xi_{B,C_{i}}{\left(\mu_{C_{i}}+\xi_{C_{i},B}\right)}^{-1}+1\right),\\ m_{C_{i}}={\left(\mu_{C_{i}}+\xi_{C_{i},B}\right)}^{-1}\left(\xi_{C_{i},B}m_{B}+1\right),\;i\in \left\{1,\ldots ,M\right\},\\ {\hat{m}}_{B}={\left(\mu_{B}+\overset{M}{\underset{i=1}{\sum }}\xi_{B,C_{i}}-\overset{M}{\underset{i=1}{\sum }}\xi_{B,C_{i}}\xi_{C_{i},B}{\left(\mu_{C_{i}}+\xi_{C_{i},B}\right)}^{-1}\right)}^{-1}\\ \;\times \left(\overset{M}{\underset{i=1}{\sum }}\xi_{B,C_{i}}\lambda_{C_{i}}{\left(\mu_{C_{i}}+\xi_{C_{i},B}\right)}^{-1}+\lambda_{B}\right),\\ {\hat{m}}_{C_{i}}={\left(\mu_{C_{i}}+\xi_{C_{i},B}\right)}^{-1}\left(\xi_{C_{i},B}{\hat{m}}_{B}+\lambda_{C_{i}}\right),\;i\in \left\{1,\ldots ,M\right\}. \end{array}$$
  We note that although we only report here expressions for the mean values, the Laplace-Stieltjes transform of *T*_*i*_, as well as the complete probability mass function of *N*_*i*_, can be explicitly obtained.
- It is clear that the number of division events can be split as $N_{i}=N_{i}\left(B\right)+\overset{M}{\underset{k=1}{\sum }}N_{i}\left(C_{k}\right)$, where *N*_*i*_(*j*) is the number of division events of a given cell taking place in the spatial compartment *j* ∈ {*B, C*_1_, …, *C*_*M*_}. The mean values (and the complete probability mass function, see section 1 in the Appendix) of these random variables *N*_*i*_(*j*), ${\hat{m}}_{i}\left(j\right)=\text{𝔼}\left(N_{i}\left(j\right)\right)$, can also be analytically computed:
  $$\begin{array}{l} {\hat{m}}_{B}(B)\text{​​}=\text{ ​​}{\left(\mu_{B}\text{​​}+\text{​​}\sum_{i=1}^{M}\xi_{B,C_{i}}\text{​}-\text{​}\sum_{i=1}^{M}\xi_{B,C_{i}}{\left(\mu_{C_{i}}\text{​}+\text{​}\xi_{C_{i},B}\right)}^{-1}\xi_{C_{i},B}\right)}^{-1}\text{​}\lambda_{B}\;,\\ {\hat{m}}_{C_{i}}(B)={\left(\mu_{C_{i}}+\xi_{C_{i},B}\right)}^{-1}\xi_{C_{i},B}{\hat{m}}_{B}(B)\;,\;i\in \{1,\ldots ,M\}\;,\\ {\hat{m}}_{B}(C_{j})={\left(\mu_{B}+\sum_{i=1}^{M}\xi_{B,C_{i}}-\sum_{i=1}^{M}\xi_{B,C_{i}}{\left(\mu_{C_{i}}+\xi_{C_{i},B}\right)}^{-1}\xi_{C_{i},B}\right)}^{-1}\\ \;\times \;\frac{\xi_{B,C_{j}}\lambda_{C_{j}}}{\mu_{C_{j}}+\xi_{C_{j},B}}\;,\;j\in \{1,\ldots ,M\}\;,\\ {\hat{m}}_{C_{i}}(C_{j})={\left(\mu_{C_{i}}+\xi_{C_{i},B}\right)}^{-1}(\xi_{C_{i},B}{\hat{m}}_{B}(C_{j})+1_{i=j}\lambda_{C_{i}}),\\ \;i,j\in \{1,\ldots ,M\}\;, \end{array}$$
  where 1_*A*_ is a function equal to 1 if *A* is satisfied, and equal to 0 otherwise.
- One can identify the spatial compartment where the cell dies, in terms of the following probabilities
  $$\begin{array}{l} \beta_{i}\left(j\right)=\text{“probability that the cell starting in compartment i},\\ {\text{ dies in compartment}j}^{\prime \prime }\\ \;=\mathbb{P}\left(Y\left(T_{i}\right)=j\right),\;i,j\in \left\{B,C_{1},\ldots ,C_{M}\right\}. \end{array}$$
  These probabilities are given by
  $$\begin{array}{l} \beta_{B}\left(B\right)={\left(\mu_{B}+\overset{M}{\underset{i=1}{\sum }}\xi_{B,C_{i}}-\overset{M}{\underset{i=1}{\sum }}\xi_{B,C_{i}}{\left(\mu_{C_{i}}+\xi_{C_{i},B}\right)}^{-1}\xi_{C_{i},B}\right)}^{-1}\mu_{B},\\ \beta_{C_{i}}\left(B\right)={\left(\mu_{C_{i}}+\xi_{C_{i},B}\right)}^{-1}\xi_{C_{i},B}\beta_{B}\left(B\right),\;i\in \left\{1,\ldots ,M\right\},\\ \beta_{B}\left(C_{j}\right)={\left(\mu_{B}+\overset{M}{\underset{i=1}{\sum }}\xi_{B,C_{i}}-\overset{M}{\underset{i=1}{\sum }}\xi_{B,C_{i}}\xi_{C_{i},B}{\left(\mu_{C_{i}}+\xi_{C_{i},B}\right)}^{-1}\right)}^{-1}\\ \;\times \frac{\xi_{B,C_{j}}\mu_{C_{j}}}{\mu_{C_{j}}+\xi_{C_{j},B}},\;j\in \left\{1,\ldots ,M\right\},\\ \beta_{C_{i}}\left(C_{j}\right)={\left(\mu_{C_{i}}+\xi_{C_{i},B}\right)}^{-1}\left(\xi_{C_{i},B}\beta_{B}\left(C_{j}\right)+\mu_{C_{i}}1_{i=j}\right),\\ \;i,j\in \left\{1,\ldots ,M\right\}. \end{array}$$
- Finally, the summary statistics introduced above refer to a single cell, without keeping track of the daughters produced by cell division. To this end, one can analyse for a given original cell in Figure 1 its complete genealogy in terms of the random variable
  $$\begin{array}{l} G_{i}=“total\;number\;of\;cells\;within\;the\;genealogy\;of\;a\;cell\\ \;{\text{which starts in compartment }i}^{\prime \prime }, \end{array}$$
  and the mean values of these random variables, ${\tilde{m}}_{i}=\text{𝔼}\left(G_{i}\right)$, can be computed as
  $$\begin{array}{l} {\tilde{m}}_{B}=(\mu_{B}+\sum_{i=1}^{M}\xi_{B,C_{i}}-\lambda_{B}-\sum_{i=1}^{M}\xi_{B,C_{i}}{\left(\mu_{C_{i}}+\xi_{C_{i},B}-\lambda_{C_{i}}\right)}^{-1}\\ \;\times \;\xi_{C_{i},B}{)}^{-1}\text{​​​}\left(\sum_{i=1}^{M}\xi_{B,C_{i}}{\left(\mu_{C_{i}}+\xi_{C_{i},B}-\lambda_{C_{i}}\right)}^{-1}2\lambda_{C_{i}}\text{​}+\text{​}2\lambda_{B}\right)\text{​},\\ {\tilde{m}}_{C_{i}}={\left(\mu_{C_{i}}+\xi_{C_{i},B}-\lambda_{C_{i}}\right)}^{-1}(\xi_{C_{i},B}{\tilde{m}}_{B}+2\lambda_{C_{i}}),\\ \;i\in \{1,\ldots ,M\}\;. \end{array}$$
  For a particular realization of the stochastic process described by Figure 1, we show in Figure 2, the definition of this summary statistics.
  We note that if on average, a larger number of division events take place than death ones, the corresponding branching process depicted in Figure 2 might explode. This means that, depending on the parameter values, one might have ℙ(*G*_*i*_ = +∞) > 0 and thus, IE(*G*_*i*_) = +∞. We find that sufficient conditions on the parameters to ensure ℙ(*G*_*i*_ = +∞) = 0, are given by
  (1) $$\begin{array}{lll} \xi_{C_{i},B}+\mu_{C_{i}} & > & \lambda_{C_{i}},\;\forall i\in \left\{1,\ldots ,M\right\}, \end{array}$$
  (2) $$\begin{array}{lll} \overset{M}{\underset{i=1}{\sum }}\xi_{B,C_{i}}+\mu_{B} & > & \lambda_{B}+\overset{M}{\underset{i=1}{\sum }}\frac{\xi_{B,C_{i}}\xi_{C_{i},B}}{\xi_{C_{i},B}+\mu_{C_{i}}-\lambda_{C_{i}}}. \end{array}$$
  We also note that there is an intuitive interpretation of these conditions. In particular, for each spatial compartment *C*_*i*_, the total rate of removing cells from this compartment (*migration* of cells, ξ_*C*_*i*_, *B*_, or death, μ_*C*_*i*__) needs to be larger than the corresponding division rate λ_*C*_*i*__, so that cells do not indefinitely accumulate in this compartment. This is represented by Equation (1). On the other hand, it is not enough to export these cells to a different compartment if these cells cannot die sufficiently fast in a different compartment after they migrate, which is summarized by Equation (2), where blood acts as a special migration hub.

### 2.3. Fast-Migration Approximation

As we show in section 3 for CD4^+^ T cells in mice, the migration rates, {(ξ_*B*,*C*_*i*__, ξ_*C*_*i*_, *B*_), *i* ∈ {1, …, *M*}}, are of the order of *min*^−1^, and division, (λ_*B*_, λ_*C*_1__, …, λ_*C*_*M*__), and death rates, (μ_*B*_, μ_*C*_1__, …, μ_*C*_*M*__), are of the order of *days*^−1^. Thus, migration is several orders of magnitude faster. One can use this fact to propose a fast-migration approximation for the summary statistics above, and thus, to study a much simpler birth-and-death (or branching) process without spatial compartments.

We propose to approximate the journey of the cell under analysis, and its progeny, by considering a birth-and-death stochastic process within a single spatial compartment, with birth and death rates given by

$$\begin{array}{lll} \bar{\lambda } & = & f_{B}\lambda_{B}+\overset{M}{\underset{i=1}{\sum }}f_{C_{i}}\lambda_{C_{i}},\;\bar{\mu }\;=\;f_{B}\mu_{B}+\overset{M}{\underset{i=1}{\sum }}f_{C_{i}}\mu_{C_{i}}, \end{array}$$

where *f*_*j*_ represents the fraction of time that the cell under study spends in each spatial compartment *j* ∈ {*B, C*_1_, …, *C*_*M*_}, in the absence of division and death (i.e., if only migration is considered in Figure 1). One could imagine that this birth-and-death process would approximate well the division and death dynamics of the original one when migration occurs at a much faster rate than division and death, so that steady state conditions (i.e., *f*_*i*_ values) for the spatial location of the cell can be assumed before any division or death event occurs.

In order to compute the fraction *f*_*j*_, one needs to calculate the steady state probabilities for the process in Figure 1, in the absence of division and death, which satisfy the following system of equations

$$\begin{array}{r} \left(\begin{array}{l} f_{B}\;f_{C_{1}}\;\ldots \;f_{C_{M}} \end{array}\right)\left(\begin{array}{ccccc} -\sum_{i}\xi_{B,C_{i}} & \xi_{B,C_{1}} & \xi_{B,C_{2}} & \ldots & \xi_{B,C_{M}}\\ \xi_{C_{1},B} & -\xi_{C_{1},B} & 0 & \ldots & 0\\ \xi_{C_{2},B} & 0 & -\xi_{C_{2},B} & \ldots & 0\\ \vdots & \vdots & \vdots & \ddots & \vdots \\ \xi_{C_{M},B} & 0 & 0 & \ldots & -\xi_{C_{M},B} \end{array}\right)=0,\\ \left(\begin{array}{l} f_{B}\;f_{C_{1}}\;\ldots \;f_{C_{M}} \end{array}\right)\left(\begin{array}{c} 1\\ 1\\ \vdots \\ 1 \end{array}\right)=1, \end{array}$$

which leads to the solution

(3) $$\begin{array}{lll} f_{B} & = & \frac{1}{1+\overset{M}{\underset{i=1}{\sum }}K_{i}},\;f_{C_{j}}\;=\;K_{j}\frac{1}{1+\overset{M}{\underset{i=1}{\sum }}K_{i}},\;j\in \left\{1,\ldots ,M\right\}, \end{array}$$

where we have introduced

$$\begin{array}{lll} K_{i} & = & \frac{\xi_{B,C_{i}}}{\xi_{C_{i},B}},\;i\in \left\{1,\ldots ,M\right\}. \end{array}$$

Once this birth-and-death approximation has been introduced, one can propose the following simplifications:

- The average lifetime of the cell in the original model can be approximated by $\text{𝔼}\left(T_{i}\right)\approx {\bar{\mu }}^{-1}$, which is its average lifetime in the fast-migration approximation, and does not depend on the initial compartment *i*. From now on, and when implementing the fast-migration approximation, we remove the initial compartment (labeled by *i*) in the notation.
- The average number of division events during the lifetime of a given cell in the original model can be approximated by $\text{𝔼}\left(N\right)\approx \frac{\bar{\lambda }}{\bar{\mu }}$, which is the average number of division events in the fast-migration approximation. Since $N=N\left(B\right)+\overset{M}{\underset{k=1}{\sum }}N\left(C_{k}\right)$, as explained in section 2.2, where *N*(*j*) is the number of division events in compartment *j* ∈ {*B, C*_1_, …, *C*_*M*_}, one can propose $\text{𝔼}\left(N\left(j\right)\right)\approx \frac{\bar{\lambda }}{\bar{\mu }}f_{j}$.
- The time for the progeny of a single cell to become extinct in the original process is not easy to analyse, and was not considered in section 2.2. Yet, it can be approximated by the time to extinction of a birth-and-death process with birth rate $\bar{\lambda }$ and death rate $\bar{\mu }$, for one single cell starting the process. This time follows a phase-type distribution (40), and its mean is given by $-{\bar{\lambda }}^{-1}\log\left(1-\frac{\bar{\lambda }}{\bar{\mu }}\right)$ (41).
- The probability of the original cell dying in compartment *j* ∈ {*B, C*_1_, …, *C*_*M*_} can be approximated by $\beta \left(j\right)\approx \frac{\mu_{j}f_{j}}{\bar{\mu }}$.
- The number of cells in the genealogy of a single cell in the original process can be approximated by the number of cells in the genealogy of a branching process with division rate $\bar{\lambda }$ and death rate $\bar{\mu }$. In particular, we can write (see section 1 in the Appendix for further details)
  (4) $$\begin{array}{l} \text{𝔼}\left(G\right)\approx \frac{\bar{\mu }+\bar{\lambda }}{\bar{\mu }-\bar{\lambda }}. \end{array}$$

### 2.4. The Effect of Non-blood Mediated Migration

**Figure 3:**
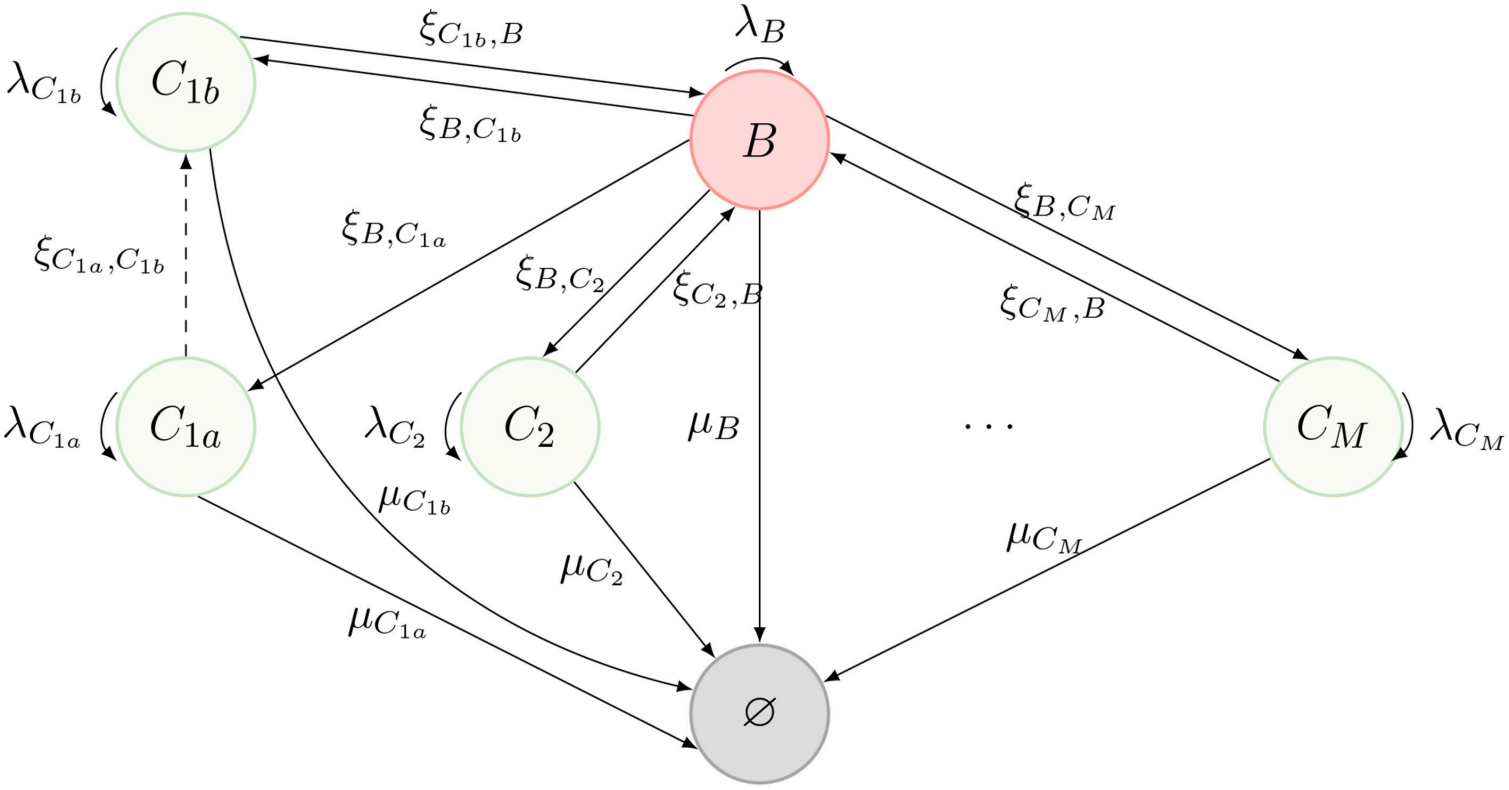
New process inspired by Ganusov and Auerbach (3, Figure 2), where T cells can migrate between two compartments (*C*_1*a*_ and *C*_1*b*_) without transitioning through the blood.

Thus far, we have considered that, as described in Figure 1, cells can only migrate from one compartment to another through blood. However, in Ganusov and Auerbach (3), the authors made a compelling case for direct migration between compartments, namely, non-mediated by blood. In this section, we make use of the same model as before, but allow for cells in compartment *C*_1*a*_ to migrate directly to compartment *C*_1*b*_. The new scenario is described in Figure 3, where the dashed arrow is the new migration rate. Note that, following Ganusov and Auerbach (3), we do not allow for migration from *C*_1*a*_ to blood.

In order to keep the notation consistent, we split the former compartment *C*_1_ into two compartments, labeled *C*_1*a*_ and *C*_1*b*_, respectively. Thus, the process of Figure 1 represents the dynamics of Figure 3, when one is not interested in deciphering where exactly a given cell is located in *C*_1_ (i.e., if the cell is in *C*_1*a*_ or *C*_1*b*_).

The summary statistics defined in section 2.2 could be analyzed for the process of Figure 3 in a similar way, but we do not present the details here. On the other hand, the fast-migration approximation can be implemented by considering the steady state migration dynamics of Figure 3, which leads to the new set of equations

$$\begin{array}{r} \left(\begin{array}{l} f_{B}\;f_{C_{1a}}\;f_{C_{1b}}\;\ldots \;f_{C_{M}} \end{array}\right)\;\\ \times \left(\begin{array}{cccccc} -\sum_{i}\xi_{B,C_{i}} & \xi_{B,C_{1a}} & \xi_{B,C_{1b}} & \xi_{B,C_{2}} & \ldots & \xi_{B,C_{M}}\\ 0 & -\xi_{C_{1a},C_{1b}} & \xi_{C_{1a},C_{1b}} & 0 & \ldots & 0\\ \xi_{C_{1b},B} & 0 & -\xi_{C_{1b},B} & 0 & \ldots & 0\\ \vdots & \vdots & \vdots & \vdots & \ddots & \vdots \\ \xi_{C_{M},B} & 0 & 0 & 0 & \ldots & -\xi_{C_{M},B} \end{array}\right)=0, \end{array}$$

$$\begin{array}{l} \left(\begin{array}{l} f_{B}\;f_{C_{1a}}\;f_{C_{1b}}\;\ldots \;f_{C_{M}} \end{array}\right)\left(\begin{array}{c} 1\\ 1\\ \vdots \\ 1 \end{array}\right)=1. \end{array}$$

One can solve this system of equations to find

(5) $$\begin{array}{l} f_{C_{1a}}=\frac{f_{B}\xi_{B,C_{1a}}}{\xi_{C_{1a},C_{1b}}},\;f_{C_{1b}}=\frac{f_{B}\left(\xi_{B,C_{1a}}+\xi_{B,C_{1b}}\right)}{\xi_{C_{1b},B}},\\ \;f_{C_{i}}=\frac{f_{B}\xi_{B,C_{i}}}{\xi_{C_{i},B}},\;i\in \left\{2,\ldots ,M\right\}. \end{array}$$

Let us introduce

$$\begin{array}{l} K_{1a}=\frac{\xi_{B,C_{1a}}}{\xi_{C_{1a},C_{1b}}},\;K_{1b}=\frac{\xi_{B,C_{1b}}}{\xi_{C_{1b},B}},\;K_{1a,1b}=\frac{\xi_{C_{1a},C_{1b}}}{\xi_{C_{1b},B}}, \end{array}$$

and $K=K_{1a}K_{1a,1b}+K_{1a}+K_{1b}+\overset{M}{\underset{i=2}{\sum }}K_{i}$, to be able to write

(6) $$\begin{array}{c} f_{B}=\frac{1}{K+1},\;f_{C_{1a}}=\frac{K_{1a}}{K+1},\;f_{C_{1b}}=\frac{K_{1a}K_{1a,1b}+K_{1b}}{K+1},\\ \;f_{C_{i}}=\frac{K_{i}}{K+1},\;i\in \left\{2,\ldots ,M\right\}. \end{array}$$

Interestingly, by adding the fractions in compartments *C*_1*a*_ and *C*_1*b*_, we can map this model to the previous one if we define

(7) $$\begin{array}{l} \xi_{B,C_{1}}=\xi_{B,C_{1a}}+\xi_{B,C_{1b}}, \end{array}$$

and

(8) $$\begin{array}{r} \frac{1}{\xi_{C_{1},B}}=\frac{\xi_{B,C_{1a}}}{\xi_{C_{1a},C_{1b}}\left(\xi_{B,C_{1a}}+\xi_{B,C_{1b}}\right)}+\frac{\xi_{B,C_{1a}}}{\xi_{C_{1b},B}\left(\xi_{B,C_{1a}}+\xi_{B,C_{1b}}\right)}\\ +\frac{\xi_{B,C_{1b}}}{\xi_{C_{1b},B}\left(\xi_{B,C_{1a}}+\xi_{B,C_{1b}}\right)}. \end{array}$$

We note that Equations (7)–(8) imply that the parameters (ξ_*C*_1_, *B*_, ξ_*B*,*C*_1__) can be considered as *effective* migration rates between the blood and compartment *C*_1_, when *C*_1_ is merged from compartments *C*_1*a*_ and *C*_1*b*_. The rate of a cell migrating from the blood to *C*_1*a*_ or *C*_1*b*_, if one is not interested in where exactly it migrates to (i.e., migration to *C*_1_), would then be given by ξ_*B*,*C*_1__ = ξ_*B*,*C*_1*a*__ + ξ_*B*,_*C*1*b*__ [see Equation (7)]. On the other hand, for a cell in *C*_1_, the mean time to reach the blood ($\text{𝔼}\left(T_{C_{1}\to B}\right)=\xi_{C_{1},B}^{-1}$) can be computed from the following analysis

$$\begin{array}{l} \xi_{C_{1},B}^{-1}=\text{𝔼}\left(T_{C_{1}\to B}\;|\;cell\;is\;at\;C_{1a}\right)\mathbb{P}\left(cell\;is\;at\;C_{1a}\right)\\ \;+\text{𝔼}\left(T_{C_{1}\to B}\;|\;cell\;is\;at\;C_{1b}\right)\mathbb{P}\left(cell\;is\;at\;C_{1b}\right). \end{array}$$

Finally, Equation (8) can be derived by noting that

$$\begin{array}{r} \mathbb{P}\left(cell\;is\;at\;C_{1a}\right)=\frac{\xi_{B,C_{1a}}}{\xi_{B,C_{1a}}+\xi_{B,C_{1b}}},\;\\ \;\mathbb{P}\left(cell\;is\;at\;C_{1b}\right)=\frac{\xi_{B,C_{1b}}}{\xi_{B,C_{1a}}+\xi_{B,C_{1b}}},\;\\ \text{𝔼}\left(T_{C_{1}\to B}\;|\;cell\;is\;at\;C_{1a}\right)=\xi_{C_{1a},C_{1b}}^{-1}+\xi_{C_{1b},B}^{-1},\\ \text{𝔼}\left(T_{C_{1}\to B}\;|\;cell\;is\;at\;C_{1b}\right)\;=\;\xi_{C_{1b},B}^{-1}.\; \end{array}$$

## 3. Numerical Results

In this section we carry out a numerical study to illustrate our analytical results and the fast-migration approximation, to compare our analytical results with those obtained from stochastic numerical simulations, and to show how dynamics occurring in a particular compartment can have a significant impact on the whole system. We propose in section 3.1 parameter values for the process described by Figure 1, based on those considered in den Braber et al. (1) and Ganusov and Auerbach (3). In section 3.2, we compare analytical and numerical results with those obtained from our fast-migration approximation. We analyse in section 3.3 the role played by the asymmetry in the death rates of the different spatial compartments. We focus in section 3.4 on the potential impact of (not blood-mediated) migration between compartments, inspired by the model considered in Ganusov and Auerbach (3).

### 3.1. Parameters

In Table 1, we provide baseline parameter values obtained from den Braber et al. (1) and Ganusov and Auerbach (3), for the model described in Figure 1 with *M* = 5. These spatial compartments represent, according to Ganusov and Auerbach (3), the blood (*B*), mesenteric lymph nodes and Peyer's patches (*C*_1_), lung (*C*_2_), liver (*C*_3_), spleen (*C*_4_) and subcutaneous lymph nodes (*C*_5_). In order to show the goodness of the fast-migration approximation, and to show the impact of spatial asymmetry in this system, we vary in section 3.2, section 3.3 and section 3.4 the division and death rates in the different spatial compartments, so that the values μ and λ in Table 1 should be considered baseline parameters for CD4^+^ T cells according to den Braber et al. (1).

**Table 1:** Parameter values considered in the numerical study. Blood and *M* = 5 additional spatial compartments as in Ganusov and Auerbach (3, Figure 2).

**Parameter**	**References**	**Value (min^**−1**^)**
ξ_*B*,*C*_1*a*__	(3)	0.53 × 10^−2^
ξ_*B*,*C*_1*b*__	(3)	1.06 × 10^−2^
ξ_*C*_1*b*_, *B*_	(3)	0.34 × 10^−2^
ξ_*C*_1*a*_, *C*_1*b*__	(3)	0.34 × 10^−2^
ξ_*B*,*C*_1__	Equation (7)^⋆^	1.59 × 10^−2^
ξ_*C*_1_, *B*_	Equation (8)^⋆^	2.60 × 10^−3^
ξ_*B*,*C*_2__	(3)	1.83
ξ_*C*_2_, *B*_	(3)	2.17
ξ_*B*,*C*_3__	(3)	0.41
ξ_*C*_3_, *B*_	(3)	1.14
ξ_*B*,*C*_4__	(3)	5.6 × 10^−2^
ξ_*C*_4_, *B*_	(3)	7.0 × 10^−3^
ξ_*B*,*C*_5__	(3)	2.6 × 10^−2^
ξ_*C*_5_, *B*_	(3)	3.4 × 10^−3^
μ	(1)	1.478 × 10^−5^(~1/47 days^−1^)
λ	(1)	1.458 × 10^−5^(~1/48 days^−1^)

*C_1_: Peyer's patches (C_1a_) and mesenteric lymph nodes (C_1b_); C_2_: lungs; C_3_: liver; C_4_: spleen; and C_5_: subcutaneous lymph nodes. Time units: min^−1^*.

(⋆) *We have estimated these parameters combining the parameters in Ganusov and Auerbach (3) according to Equations (7)–(8)*.

We also note that the model in Ganusov and Auerbach (3) considers Peyer's patches and mesenteric lymph nodes as two different compartments (i.e., *C*_1*a*_ and *C*_1*b*_, respectively, described in section 2.4). We propose in section 3.2 and section 3.3 to merge these compartments and analyse the cellular dynamics without deciphering where exactly a given cell in *C*_1_ is (i.e., if the cell is in the Peyer's patches or in the mesenteric lymph nodes), by using Equations (7)–(8) to obtain *effective* migration rates (ξ_*B*,*C*_1__, ξ_*C*_1_, *B*_) in Table 1. In section 3.4 we carry out a numerical study of our second model, described in Figure 3, where the migration rates (ξ_*B*,*C*_1*a*__, ξ_*B*,*C*_1*b*__, ξ_*C*_1*b*_, *B*_, ξ_*C*_1*a*_, *C*_1*b*__) are those in Ganusov and Auerbach (3). Finally, we note that for all the parameter values considered in this section, Equations (1)–(2) are satisfied.

### 3.2. Gillespie Simulations, Analytic Results, and Fast-Migration Approximation

In this section we set the migration rates {(ξ_*B*,*C*_*i*__, ξ_*C*_*i*_, *B*_), *i* ∈ {1, …, 5}} as given in Table 1. In order to show the goodness of the fast-migration approximation we set

$$\begin{array}{l} \mu_{B}=R\mu ,\;\mu_{C_{i}}=R\mu ,\;i\in \left\{1,\ldots ,5\right\},\\ \lambda_{B}=\lambda ,\;\lambda_{C_{i}}=R\lambda ,\;i\in \left\{1,\ldots ,5\right\}, \end{array}$$

for varying values of *R* > 0. We note that in the rest of the paper, but this section, we set *R* = 1. *R* = 1 corresponds to a completely symmetric scenario, where all compartments have death rate μ and division rate λ. For increasing values of *R*, the scenario becomes asymmetric, where division in the blood is less likely to occur compared to division in other compartments, while death rates are still the same in all spatial compartments. However, division and death rates for large values of *R* become comparable (similar order of magnitude) to migration rates.

**Figure 4:**
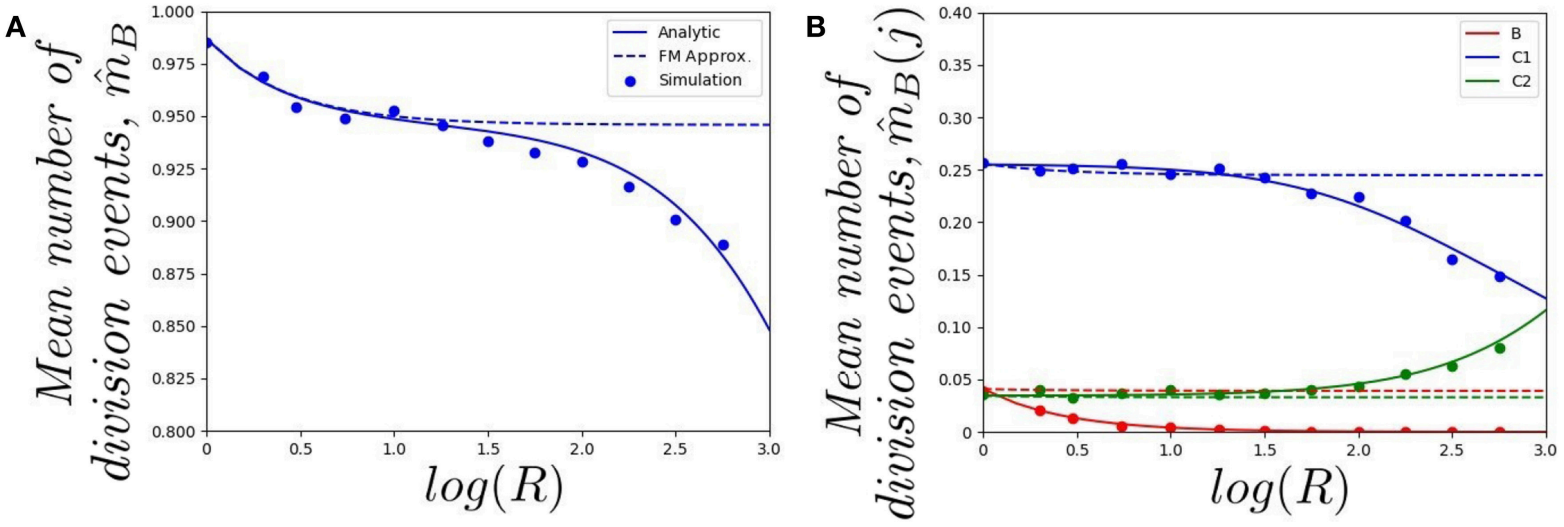
Effect of changing the value of *R* on **(A)** the mean total number of division events ${\hat{m}}_{B}$, and **(B)** the mean number of division events occurring in compartment *j*, ${\hat{m}}_{B}\left(j\right)$, for *j* ∈ {*B, C*_1_, *C*_2_}. We consider a single cell starting in the blood. Analytic solution (*solid*), fast-migration approximation (*dashed*) and 10^4^ stochastic simulations (*dots*).

In Figure 4, we plot (starting with a single cell in the blood) *a*) the mean number of division events in the blood and *b*) the mean number of division events in compartments {*B, C*_1_, *C*_2_}, as a function of log(*R*). The fast-migration (FM) approximation mostly provides reliable results when log(*R*) < 1.0. In this case, results obtained by simulations agree with the analytic results (obtained as detailed in section 2.2), and with the fast-migration approximation (computed as explained in section 2.3). We note that values log(*R*) < 1.0 correspond to division and apoptotic rates of the order of 10^−5^−10^−4^ min^−1^, and migration rates are of the order of 10^−3^−10^0^ min^−1^. For log(*R*) > 1.0, division and death rates become comparable to some of the migration rates, and the fast-migration approximation provides results in Figure 4 which significantly differ from those obtained by numerical simulations and analytic methods. We also note that even for small values of log(*R*), other variables of interest cannot be well captured by the fast-migration approximation. This is the case for ${\hat{m}}_{B}\left(B\right)=\text{𝔼}\left(N_{B}\left(B\right)\right)$, the mean number of division events occurring in the blood for a cell starting in the blood. While the fast-migration approximation provides reliable results for log(*R*) = 0, once log(*R*) > 0 the true (analytic) value of ${\hat{m}}_{B}\left(B\right)$ fastly decays to zero. This behavior is captured by the stochastic simulations in Figure 4B, but not by the fast-migration approximation.

**Figure 5:**
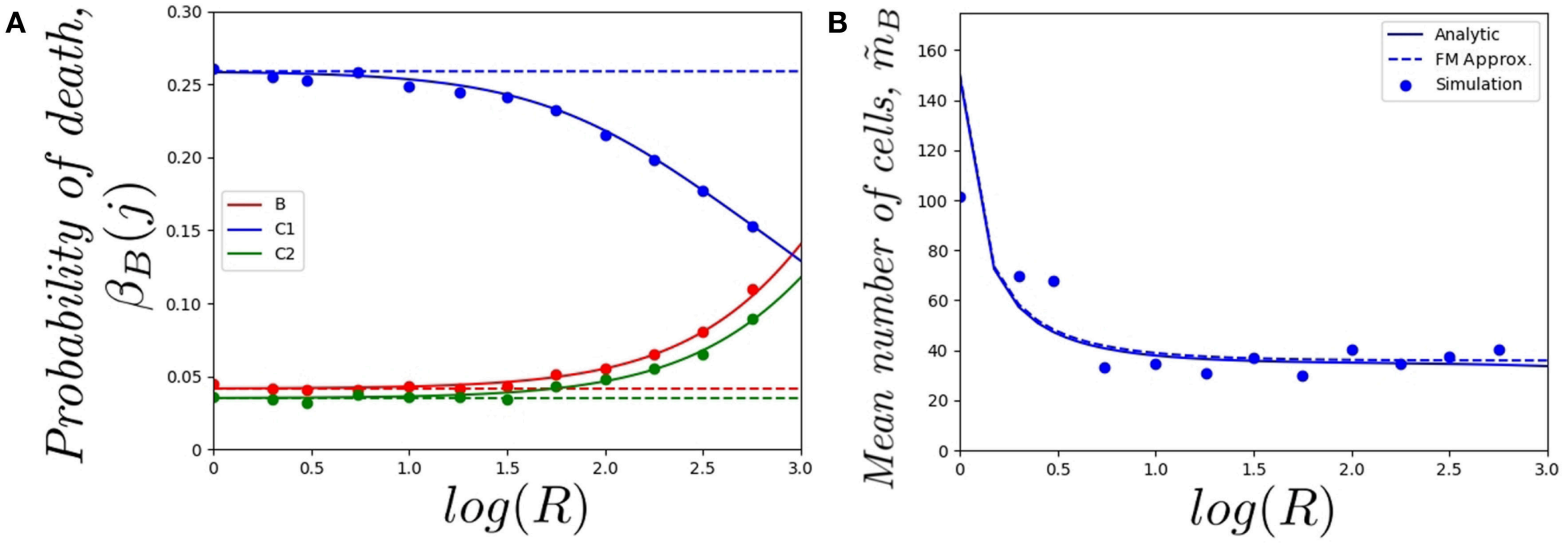
Effect of changing the value of *R* on **(A)** the probability of the cell dying in compartment *j*, for *j* ∈ {*B, C*_1_, *C*_2_}, and **(B)** the mean total number of cells in the genealogy of the original cell, ${\tilde{m}}_{B}=\text{E}\left(G_{B}\right)$. We consider a single cell starting in the blood. Analytic result (*solid*), fast-migration approximation (*dashed*) and 10^4^, for **(A)**, and 10^3^, for **(B)**, stochastic simulations (*dots*).

In Figure 5, we plot *a*) the probability of the original cell dying in compartments {*B, C*_1_, *C*_2_}, and *b*) the mean total number of cells in the genealogy of the original cell, as a function of log(*R*) and for a cell starting in the blood. Similar comments to the ones above apply to the results in Figure 5A, where the fast-migration approximation behaves well for log(*R*) < 1.0. We also note that for small values of *R* we get β_*B*_(*C*_1_) > β_*B*_(*B*) > β_*B*_(*C*_2_) for the cell starting in the blood, which shows the importance of the migration dynamics in the fate of a given cell. However, the probability of the cell dying in the blood increases with increasing values of *R* as one would expect, since if division and death rates increase, the starting position of the cell has a larger impact on its proliferation and death dynamics and the impact of migration rates accordingly decreases. It is also interesting to note that the fast-migration approximation provides reliable results for all the values of *R* explored in Figure 5B. In this case, we study the mean number of cells in the genealogy, which is a population-based descriptor rather than a descriptor only related to a given cell. This result is striking, since division and death rates for log(*R*) > 2.0 are of the order of 10^−3^−10^−2^ min^−1^, which are comparable to the migration rates in Table 1. This might indicate that the fast-migration approximation could behave better when dealing with population-based summary statistics, while it is reliable when analysing single cell descriptors only for small enough values of the division and death rates (compared to migration rates).

It is also worth noting that simulating stochastic processes with different timescales is a challenging problem from a computational point of view, which in our case means simulating the migration of cells (timescales of the order of minutes), until some division or death events occur (in days). This is even more challenging when dealing with a population of cells (see Figure 5B) rather than a single cell (Figures 4, 5A). For these computational reasons, 10^3^ stochastic simulations were used to compute the values in Figure 5B, compared to 10^4^ simulations for Figures 4, 5A. Thus, our results in Figure 5B illustrate the need for developing the analytical results of section 2.2, or related approximations, such as the one in section 2.3, instead of using standard stochastic simulations to analyse cellular dynamics in these systems.

### 3.3. The Role of Different Death Rates in Different Compartments

We have assumed that death rates are the same in all compartments. However, taking into account that migration rates determine the relative weight of each compartment in the overall behavior of the system, in this section we analyse the role of different death rates in compartments *C*_1_ (where migration from blood is around six times faster than the reverse) and *C*_3_ (where migration from blood is around one third slower than the reverse) (3).

**Figure 6:**
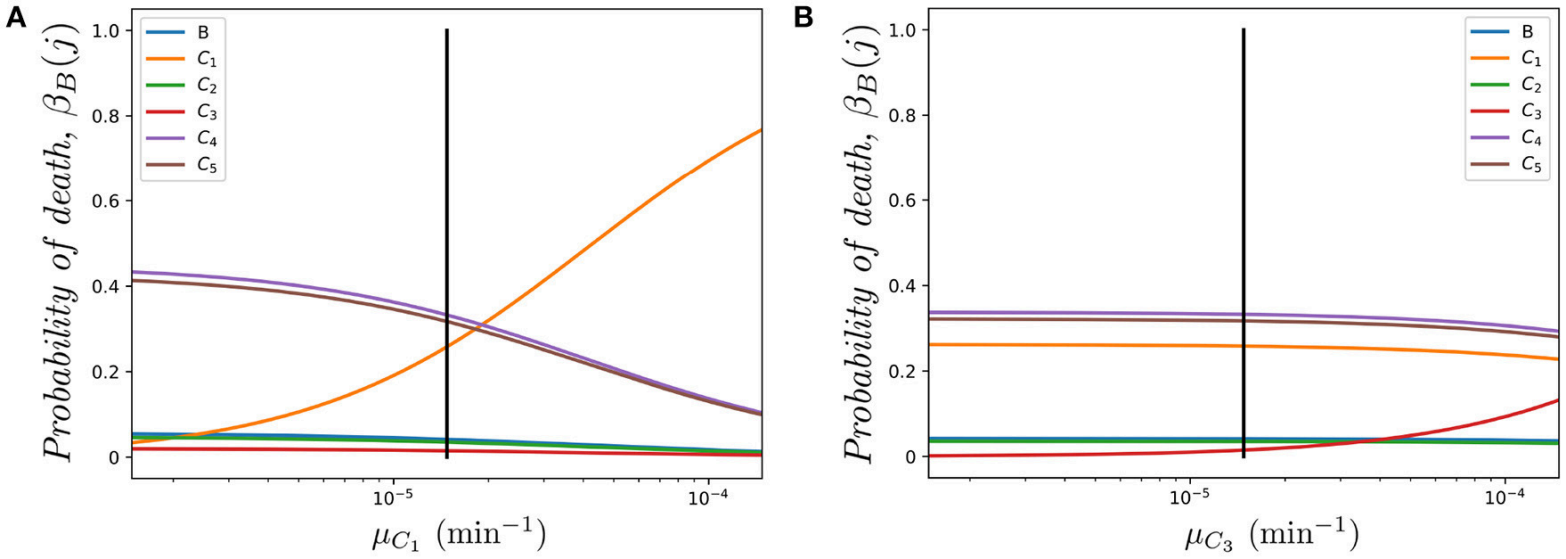
Effect of varying μ_*C*_*i*__ on the probability β_*B*_(*j*) of dying in different compartments *j* ∈ {*B, C*_1_, …, *C*_5_}, for a cell starting at blood. **(A)** Varying μ_*C*_1__; **(B)** Varying μ_*C*_3__. The vertical black line represents the value μ.

Figure 6A shows the impact of varying the death rate μ_*C*_1__ in compartment *C*_1_ (with respect to the one in Table 1 μ_*C*_1__ = μ, shown as a vertical solid line). It is interesting to note how compartment *C*_4_ is one of the most sensitive to this parameter in spite of the fact that we are only changing the death rate of compartment *C*_1_. The rationale behind this result is related to the relative *immigration* to *emigration* rates in each compartment. In particular, using the migration rates of Table 1 we find *K*_1_ = 6.115, *K*_2_ = 0.843, *K*_3_ = 0.360, *K*_4_ = 8.000, *K*_5_ = 7.647 (namely, immigration in *C*_1_ is 6.115 times larger than emigration). The three compartments with higher immigration/emigration ratios are *C*_1_, *C*_4_ and *C*_5_. Thus, when μ_*C*_1__ increases, the weight of the dynamics is shifted to the compartment with the highest such ratio. On the other hand, in Figure 6B, as compartment *C*_3_ has low *K*_3_ = 0.360, the most sensitive probability of death with respect to parameter μ_*C*_3__ is β_*B*_(*C*_3_).

**Figure 7:**
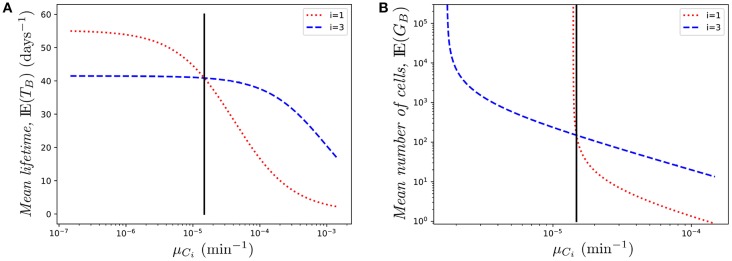
Effect of changing the value of one death rate, μ_*C*_*i*__ on **(A)** the mean lifetime, IE(*T*_*B*_), and **(B)** the mean number of cells in the genealogy, IE(*G*_*B*_), for a cell starting at blood. The vertical black line represents the value μ.

Similarly, Figure 7A shows the effect of changing μ_*C*_1__ on the mean lifetime of a cell. Again, the role of *K*_*i*_ is very relevant. While in both cases (changing μ_*C*_1__ or μ_*C*_3__) the mean lifetime decreases (as expected), the effect is milder in the case of μ_*C*_3__. This is related to the fact that, due to the reduced immigration rate into compartment *C*_3_, the odds of finding the cell in that compartment are relatively low. On the contrary, as the cell spends more time in compartment *C*_1_, varying the death rate in that compartment increases/decreases quickly the mean lifetime when the death rate decreases/increases, respectively.

Finally, in Figure 7B we show the mean number of cells produced by a single cell during its lifetime (the size of the offspring tree). Again, in the case of compartment *C*_1_, reducing slightly the death rate produces a transition from finite to infinite number of descendants, which is directly related to the conditions given by Equations (1)–(2), and relates to the asymptotic behavior observed in Figure 7B. In this case, small changes in the parameters might have a huge impact on the cell population dynamics.

Overall, these analyses show that, not only migration rates, but the ratio between immigration and emigration rates affect the overall dynamics of the system. Thus, analysing these ratios, and their interplay with the apoptotic and proliferation rates, can help to identify the most relevant locations of the immune system related to the fate of a single cell.

### 3.4. The Role of Direct Migration Between Compartments

**Figure 8:**
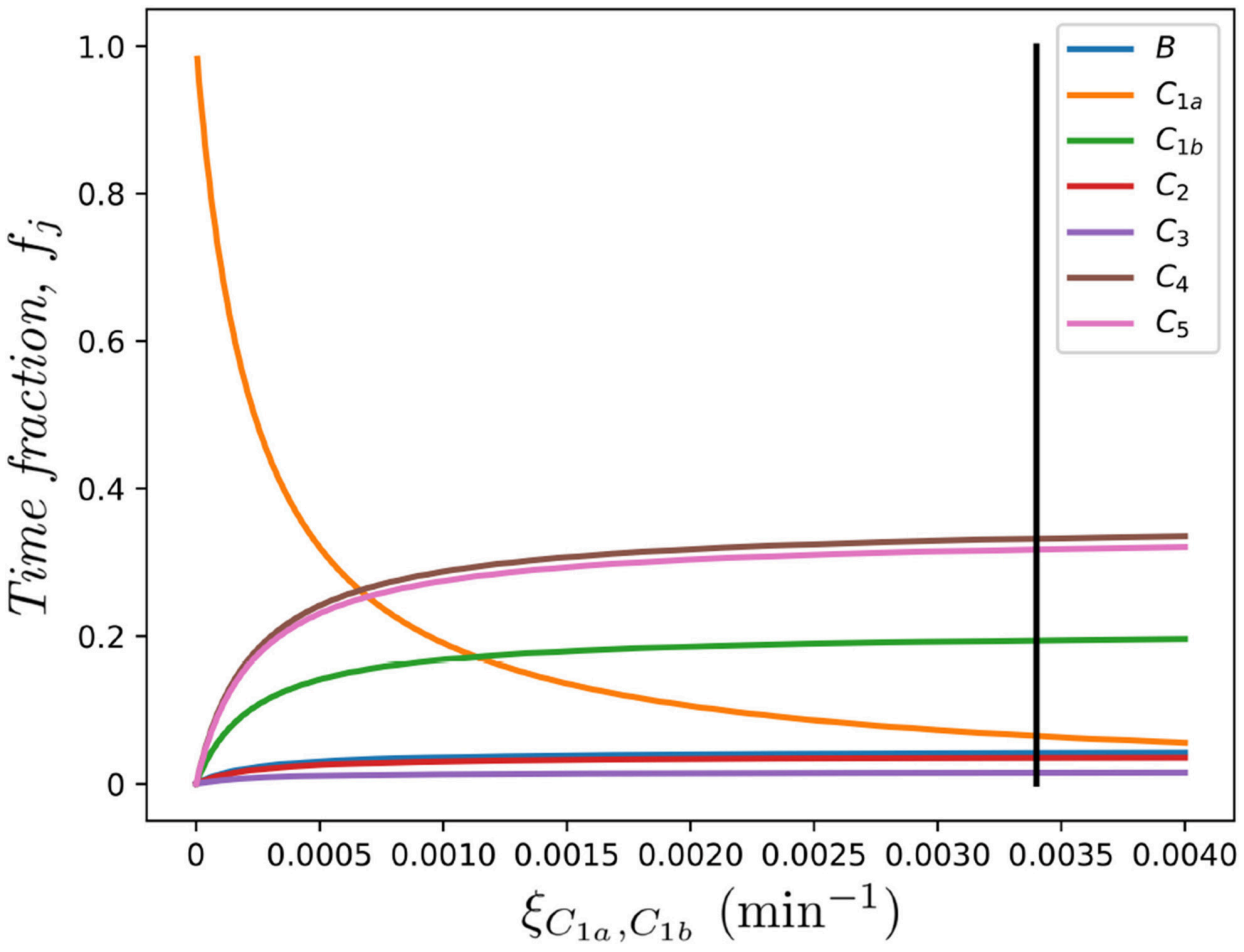
Effect of the migration rate ξ_*C*_1*a*_, *C*_1*b*__ on the fraction of time spent in each compartment, *f*_*j*_, for *j* ∈ {*B, C*_1*a*_, *C*_1*b*_, *C*_2_, …, *C*_5_}. The vertical black line represents the value ξ_*C*_1*a*_, *C*_1*b*__ in Table 1, as in Ganusov and Auerbach (3).

To test the relevance of migration between compartments (as emphasized in Ganusov and Auerbach (3)) we compute the fractions, *f*_*B*_, *f*_*C*_1*a*__, …, *f*_*C*_5__ using the parameters from Table 1. We have

$$\begin{array}{r} f_{B}=0.042,\;f_{C_{1}}=0.255,\;f_{C_{2}}=0.035,\;f_{C_{3}}=0.015,\\ \;f_{C_{4}}=0.334,\;f_{C_{5}}=0.319, \end{array}$$

where, as we defined above, *f*_*j*_ represents the fraction of time that the cell under study spends in each spatial compartment *j* ∈ {*B, C*_1_, …, *C*_*M*_}, in the absence of division and death. Furthermore, in Figure 8 we show the dependence of these fractions on the rate connecting compartments *C*_1*a*_ and *C*_1*b*_, ξ_*C*_1*a*_, *C*_1*b*__. Clearly, as ξ_*C*_1*a*_, *C*_1*b*__ → 0, compartment *C*_1*a*_ becomes a *sink* of cells so that *f*_*C*_1*a*__ → 1 and the rest tend to 0.

### 3.5. Sensitivity Analysis

For the original model in Figure 1, we can use Equation (3) to compute the sensitivity matrix **S**. Using the parameters in Table 1, we find that the matrix **S** is given by

$$\left(\begin{array}{cccccccccc} -6.7\cdot 10^{-1} & 4.1 & -8.\cdot 10^{-4} & 6.8\cdot 10^{-4} & -1.5\cdot 10^{-3} & 5.5\cdot 10^{-4} & -2.5\cdot 10^{-1} & 2.0 & -5.1\cdot 10^{-1} & 3.9\\ 1.2\cdot 10^{1} & -7.3\cdot 10^{1} & -4.9\cdot 10^{-3} & 4.1\cdot 10^{-3} & -9.3\cdot 10^{-3} & 3.4\cdot 10^{-3} & -1.5 & 1.2\cdot 10^{1} & -3.1 & 2.4\cdot 10^{1}\\ -5.6\cdot 10^{-1} & 3.5 & 1.9\cdot 10^{-2} & -1.6\cdot 10^{-2} & -1.3\cdot 10^{-3} & 4.6\cdot 10^{-4} & -2.1\cdot 10^{-1} & 1.7 & -4.3\cdot 10^{-1} & 3.3\\ -2.4\cdot 10^{-1} & 1.5 & -2.9\cdot 10^{-4} & 2.4\cdot 10^{-4} & 3.6\cdot 10^{-2} & -1.3\cdot 10^{-2} & -8.9\cdot 10^{-2} & 7.2\cdot 10^{-1} & -1.8\cdot 10^{-1} & 1.4\\ -5.4 & 3.3\cdot 10^{1} & -6.4\cdot 10^{-3} & 5.4\cdot 10^{-3} & -1.2\cdot 10^{-2} & 4.4\cdot 10^{-3} & 4.0 & -3.2\cdot 10^{1} & -4.1 & 3.1\cdot 10^{1}\\ -5.1 & 3.1\cdot 10^{1} & -6.1\cdot 10^{-3} & 5.2\cdot 10^{-3} & -1.2\cdot 10^{-2} & 4.2\cdot 10^{-3} & -1.9 & 1.5\cdot 10^{1} & 8.4 & -6.4\cdot 10^{1} \end{array}\right),$$

where the rows stand for *f*_*B*_, *f*_*C*_1__, …, *f*_*C*_5__ and the columns for ξ_*B*,*C*_1__, ξ_*C*_1_, *B*_, …, ξ_*C*_5_, *B*_. That is, matrix **S** is defined so that

$${\text{S}}_{12}=\frac{\partial f_{B}}{\partial \xi_{C_{1},B}}\;{\text{S}}_{21}=\frac{\partial f_{C_{1}}}{\partial \xi_{B,C_{1}}},\ldots .$$

While this matrix is informative, it does not reflect a property of Equation (3); namely, that the relevant quantities are not migration rates themselves but, rather, the *immigration*/*emigration* ratio for each spatial compartment. Thus, one can obtain a simpler version of the sensitivity matrix with respect to the ratios *K*_*i*_, *i* ∈ {1, …, 5}:

$$\tilde{\text{S}}=\left(\begin{array}{lllll} -1.7\times 10^{-3} & -1.7\times 10^{-3} & -1.7\times 10^{-3} & -1.7\times 10^{-3} & -1.7\times 10^{-3}\\ +3.1\times 10^{-2} & -1.1\times 10^{-2} & -1.1\times 10^{-2} & -1.1\times 10^{-2} & -1.1\times 10^{-2}\\ -1.5\times 10^{-3} & +4.0\times 10^{-2} & -1.5\times 10^{-3} & -1.5\times 10^{-3} & -1.5\times 10^{-3}\\ -6.3\times 10^{-4} & -6.3\times 10^{-4} & +4.1\times 10^{-2} & -6.3\times 10^{-4} & -6.3\times 10^{-4}\\ -1.4\times 10^{-2} & -1.4\times 10^{-2} & -1.4\times 10^{-2} & +2.8\times 10^{-2} & -1.4\times 10^{-2}\\ -1.3\times 10^{-2} & -1.3\times 10^{-2} & -1.3\times 10^{-2} & -1.3\times 10^{-2} & +2.8\times 10^{-2} \end{array}\right),$$

where each row corresponds to a given fraction, *f*_*j*_, and with the columns representing (*K*_1_, *K*_2_, *K*_3_, *K*_4_, *K*_5_), where *K*_1_ = 6.115, *K*_2_ = 0.843, *K*_3_ = 0.360, *K*_4_ = 8.000, *K*_5_ = 7.647, so that

$${\tilde{S}}_{ij}=\{\begin{array}{cc} \frac{\partial f_{B}}{\partial K_{j}} & \text{if }i=1\;,\\ \frac{\partial f_{C_{i-1}}}{\partial K_{j}} & \text{if }i\neq 1\;. \end{array}$$

Due to some symmetries in Equation (3) with respect to *K*_*i*_, the reduced sensitivity matrix has many repeated entries. Biologically, this is a remarkable result as it shows that events occurring in different compartments can affect equally a given one. Another conclusion can be derived from the sign of the elements of $\tilde{\text{S}}$. These signs can be easily understood by noting that, since *K*_*i*_ represents the fraction of *immigration* to *emigration* events in a given compartment, the higher *K*_*i*_ the higher the probability of finding a cell in compartment *C*_*i*_.

## 4. Discussion and Conclusions

We propose a mathematical model for the migration, proliferation and death of a CD4^+^ T cell, and focus on a number of observables which refer to the single cell journey during its lifetime, as well as to the dynamics of its progeny. We have presented analytical methods to study these observables and have provided conditions for this cellular system not to explode. A fast-migration approximation can be proposed when migration events occur significantly faster than division and death, so that steady state conditions can be assumed for the spatial location of the cell.

Our numerical results show that most of the stochastic observables under study can be properly captured by means of the fast-migration approximation, when migration rates are (at least) one order of magnitude larger than division and death rates. The fast-migration approximation is able to appropriately capture the mean number of cells in the genealogy of a given cell, even when migration events occur at a similar rate to those of division and death events. It is also worth mentioning that our numerical results illustrate how perturbing a single rate in a given spatial compartment can have a significant impact on the cellular dynamics and observables corresponding to other compartments, which indicates the clear interplay between cellular dynamics in the different spatial compartments, and which depends on the specific migration structure (and rates) of the system. Finally, a particular feature of our analytical approach is that it allows for an exact sensitivity analysis of each of the observables with respect to each kinetic rate. In this way the contribution that each particular rate (i.e., event) has on a given stochastic observable can be assessed.

Recent experimental advances allow us to observe biological processes at the single cell level, but at the same time these new experimental techniques are still far from being able to provide a full picture of migration, division and death events *in vivo*. Thus, experimental observations need to be combined with mathematical and computational models which allow one to test hypotheses, shed some light on cellular dynamics or to design new or different experiments. As a consequence, two main challenges that directly arise are: (i) to develop new analytical techniques to study different observables in these cellular systems, which can be compared to experimental measurements, and (ii) to propose new and more advanced methodologies for calibrating these mathematical models by using experimental data. Although our focus in this work is on (i), our results can have a direct impact on (ii), since the distributions and mean values computed in section 2 could be used to calculate the likelihood function when applying Bayesian statistical methods for parameter estimation and model calibration.

Finally, it is worth noting that, unlike population dynamics models (based on collective counts of cells), our stochastic descriptors have two clear advantages. First of all, they represent variables directly related to the dynamics of a single cell, and thus, allow us to bridge between the novel experimental techniques described in section 2.2 and specific proliferation, death or migration rates at the cell level. Secondly, our descriptors allow us to quantify these individual rates. For instance, in practice only the net division rate can be measured but, combining descriptors, we can separate birth from death or migration rates. Naturally, these estimates require the use of both more sophisticated experiments and parameter estimation techniques (such as the Bayesian methods mentioned above).

## Author Contributions

ML-G, MC, and GL designed and analyzed the summary statistics and the fast-migration approximation. ML-G, MC, NA, and LdlH wrote the numerical codes and performed the numerical simulations. ML-G, MC, LdlH, GL, and CM-P contributed to writing the manuscript. All authors contributed to the development of the mathematical model and reviewing the literature and to revising the manuscript.

### Conflict of Interest Statement

The authors declare that the research was conducted in the absence of any commercial or financial relationships that could be construed as a potential conflict of interest.

## References

[1] den Braber I Mugwagwa T Vrisekoop N Westera L Mögling R de Boer AB Maintenance of peripheral naive T cells is sustained by thymus output in mice but not humans. Immunity. (2012) 36:288–97. 10.1016/j.immuni.2012.02.006 22365666

[2] Thome JJ Yudanin N Ohmura Y Kubota M Grinshpun B Sathaliyawala T. Spatial map of human T cell compartmentalization and maintenance over decades of life. Cell. (2014) 159:814–28. 10.1016/j.cell.2014.10.026 25417158 PMC4243051

[3] Ganusov VV Auerbach J. Mathematical modeling reveals kinetics of lymphocyte recirculation in the whole organism. PLoS Comput Biol. (2014) 10:e1003586. 10.1371/journal.pcbi.1003586 24830705 PMC4022467

[4] Herzenberg LA Parks D Sahaf B Perez O Roederer M Herzenberg LA. The history and future of the fluorescence activated cell sorter and flow cytometry: a view from Stanford. Clin Chem. (2002) 48:1819–27. Available online at: http://clinchem.aaccjnls.org/content/clinchem/48/10/1819.full.pdf 12324512

[5] Johnson J Nowicki MO Lee CH Chiocca EA Viapiano MS Lawler SE. Quantitative analysis of complex glioma cell migration on electrospun polycaprolactone using time-lapse microscopy. Tiss Eng C Methods. (2009) 15:531–40. 10.1089/ten.tec.2008.0486 19199562 PMC3497888

[6] Westera L Drylewicz J Den Braber I Mugwagwa T Van Der Maas I Kwast L. Closing the gap between T-cell life span estimates from stable isotope-labeling studies in mice and humans. Blood. (2013) 122:2205–12. 10.1182/blood-2013-03-488411 23945154

[7] Gosling JP Krishnan SM Lythe G Chain B MacKay C Molina-París C A mathematical study of CD8^+^ T cell responses calibrated with human data. *arXiv preprint arXiv:180205094* (2018).

[8] Seddon B Yates AJ. The natural history of naive T cells from birth to maturity. Immunol Rev. (2018) 285:218–32. 10.1111/imr.12694 30129206

[9] Mason D. A very high level of crossreactivity is an essential feature of the T-cell receptor. Immunol Tod. (1998) 19:395–404. 10.1016/S0167-5699(98)01299-7 9745202

[10] Jenkins MK Chu HH McLachlan JB Moon JJ. On the composition of the preimmune repertoire of T cells specific for peptide-major histocompatibility complex ligands. Ann Rev Immunol. (2010) 28:275–94. 10.1146/annurev-immunol-030409-101253 20307209

[11] Itano AA Jenkins MK. Antigen presentation to naive CD4 T cells in the lymph node. Nature Immunol. (2003) 4:733–9. 10.1038/ni957 12888794

[12] Sallusto F Lenig D Förster R Lipp M Lanzavecchia A Two subsets of memory T lymphocytes with distinct homing potentials and effector functions. Nature. (1999) 402:34–8. 10.1038/35005534 10537110

[13] Masopust D Schenkel JM. The integration of T cell migration, differentiation and function. Nat Rev. Immunol. (2013) 13:309–20. 10.1038/nri3442 23598650

[14] Farber DL Yudanin NA Restifo NP. Human memory T cells: generation, compartmentalization and homeostasis. Nat Rev Immunol. (2014) 14:24. 10.1038/nri3567 24336101 PMC4032067

[15] Kumar BV Connors TJ Farber DL. Human T cell development, localization, and function throughout life. Immunity. (2018) 48:202–13. 10.1016/j.immuni.2018.01.007 29466753 PMC5826622

[16] Davis MM Brodin P. Rebooting human immunology. Ann Rev Immunol. (2018) 36:843–64. 10.1146/annurev-immunol-042617-053206 29490162 PMC6677260

[17] Blum KS Pabst R. Lymphocyte numbers and subsets in the human blood: do they mirror the situation in all organs? Immunol Lett. (2007) 108:45–51. 10.1016/j.imlet.2006.10.009 17129612

[18] Ganusov VV De Boer RJ. Do most lymphocytes in humans really reside in the gut? Trends Immunol. (2007) 28:514–8. 10.1016/j.it.2007.08.009 17964854

[19] Hogan T Gossel G Yates AJ Seddon B. Temporal fate mapping reveals age-linked heterogeneity in naive T lymphocytes in mice. Proc Natl Acad Sci USA. (2015) 112:E6917–26. 10.1073/pnas.1517246112 26607449 PMC4687551

[20] Gonçalves P Ferrarini M Molina-Paris C Lythe G Vasseur F Lim A. A new mechanism shapes the naïve CD8^+^ T cell repertoire: the selection for full diversity. Mol Immunol. (2017) 85:66–80. 10.1016/j.molimm.2017.01.026 28212502

[21] Baliu-Piqué M Kurniawan H Ravesloot L Verheij M Drylewicz J Lievaart-Peterson K. Age-related distribution and dynamics of T cells in blood and lymphoid tissues of goats. Dev Comp Immunol. (2018) 93:1–10. 10.1016/j.dci.2018.12.004 30550777

[22] Knabel M Franz TJ Schiemann M Wulf A Villmow B Schmidt B. Reversible MHC multimer staining for functional isolation of T-cell populations and effective adoptive transfer. Nat Med. (2002) 8:631. 10.1038/nm0602-631 12042816

[23] Krutzik PO Nolan GP. Fluorescent cell barcoding in flow cytometry allows high-throughput drug screening and signaling profiling. Nat Methods. (2006) 3:361. 10.1038/nmeth872 16628206

[24] Schwab SR Cyster JG. Finding a way out: lymphocyte egress from lymphoid organs. Nat Immunol. (2007) 8:1295. 10.1038/ni1545 18026082

[25] Westermann J Söllner S Ehlers EM Nohroudi K Blessenohl M Kalies K. Analyzing the migration of labeled T cells *in vivo*: an essential approach with challenging features. Lab Invest. (2003) 83:459. 10.1097/01.LAB.0000062852.80567.90 12695549

[26] Pham K Shimoni R Charnley M Ludford-Menting MJ Hawkins ED Ramsbottom K. Asymmetric cell division during T cell development controls downstream fate. J Cell Biol. (2015) 210:933–50. 10.1083/jcb.201502053 26370500 PMC4576854

[27] Sawicka M Stritesky G Reynolds J Abourashchi N Lythe G Molina-París C. From pre-DP, post-DP, SP4, and SP8 thymocyte cell counts to a dynamical model of cortical and medullary selection. Front Immunol. (2014) 5:19. 10.3389/fimmu.2014.00019 24592261 PMC3924582

[28] Artalejo J Gómez-Corral A López-García M Molina-París C. Stochastic descriptors to study the fate and potential of naive T cell clonotypes in the periphery. J Math Biol. (2017) 74:673–708. 10.1007/s00285-016-1020-6 27350044 PMC5258823

[29] Reynolds J Coles M Lythe G Molina-París C Deterministic and stochastic naïve T cell population dynamics: symmetric and asymmetric cell division. Dyn Syst. (2012) 27:75–103. 10.1080/14689367.2011.645447

[30] Donovan GM Lythe G. T-cell movement on the reticular network. J Theor Biol. (2012) 295:59–67. 10.1016/j.jtbi.2011.11.001 22100488

[31] Currie J Castro M Lythe G Palmer E Molina-París C. A stochastic T cell response criterion. J R Soc Interface. (2012) 9:2856–70. 10.1098/rsif.2012.0205 22745227 PMC3479899

[32] Huth J Buchholz M Kraus JM Mølhave K Gradinaru C Wichert Gv. TimeLapseAnalyzer: multi-target analysis for live-cell imaging and time-lapse microscopy. Comput Methods Progr Biomed. (2011) 104:227–34. 10.1016/j.cmpb.2011.06.002 21705106

[33] Klein J Leupold S Biegler I Biedendieck R Münch R Jahn D. TLM-Tracker: software for cell segmentation, tracking and lineage analysis in time-lapse microscopy movies. Bioinformatics. (2012) 28:2276–7. 10.1093/bioinformatics/bts424 22772947

[34] Hilsenbeck O Schwarzfischer M Skylaki S Schauberger B Hoppe PS Loeffler D. Software tools for single-cell tracking and quantification of cellular and molecular properties. Nat Biotechnol. (2016) 34:703. 10.1038/nbt.3626 27404877

[35] Hawkins E Markham J McGuinness L Hodgkin P. A single-cell pedigree analysis of alternative stochastic lymphocyte fates. Proc Natl Acad Sci USA. (2009) 106:13457–62. 10.1073/pnas.0905629106 19633185 PMC2715326

[36] Piltti KM Cummings BJ Carta K Manughian-Peter A Worne CL Singh K. Live-cell time-lapse imaging and single-cell tracking of *in vitro* cultured neural stem cells–Tools for analyzing dynamics of cell cycle, migration, and lineage selection. Methods. (2018) 133:81–90. 10.1016/j.ymeth.2017.10.003 29050826 PMC6166405

[37] Reinhardt RL Khoruts A Merica R Zell T Jenkins MK. Visualizing the generation of memory CD4 T cells in the whole body. Nature. (2001) 410:101. 10.1038/35065111 11242050

[38] Masopust D Vezys V Marzo AL Lefrançois L. Preferential localization of effector memory cells in nonlymphoid tissue. Science. (2001) 291:2413–7. 10.1126/science.1058867 11264538

[39] Sathaliyawala T Kubota M Yudanin N Turner D Camp P Thome JJ. Distribution and compartmentalization of human circulating and tissue-resident memory T cell subsets. Immunity. (2013) 38:187–97. 10.1016/j.immuni.2012.09.020 23260195 PMC3557604

[40] He QM Fundamentals of Matrix-Analytic Methods. Springer (2014).

[41] Matis JH Kiffe TR Stochastic Population Models: A Compartmental Perspective. Vol. 145 New York, NY: Springer Science & Business Media (2012).

