# Supplementary material for: Fate of a Naive T Cell: A Stochastic Journey
Source: Front Immunol. 2019 Mar 6;10:194. doi: 10.3389/fimmu.2019.00194 (PMC6415700; doi:10.3389/fimmu.2019.00194)
Supplement: Supplementary file 1 [file Data_Sheet_1.pdf]

Fate of a naive T cell: a stochastic journey

L. de la Higuera[1,†], M. López-García[1,*,†], M. Castro[1,2,†], N. Abourashchi[3], G. Lythe[1], C. Molina-París[1]

[1] *University of Leeds, School of Mathematics, Department of Applied Mathematics, LS2 9JT Leeds, UK.*
[2] *Universidad Pontificia Comillas, Grupo Interdisciplinar de Sistemas Complejos (GISC) and DNL, Madrid, Spain.*
[3] *University College London, Department of Statistical Science, WC1E 6BT London, UK.*
[†] *Equal contribution*

Correspondence*:
Martín López-García
m.lopezgarcia@leeds.ac.uk

Appendix

1 DERIVATION OF THE SINGLE CELL DESCRIPTORS

1.1 Lifetime of a single cell and number of division events during its lifetime

In this section, our aim is to analyse the random variable T_i, as defined in Section 2.2 for $i \in \{B, C_1, \ldots, C_M\}$. We note that when a division event takes place one of the daughter cells remains as the marked cell; that is, when a division occurs, we consider that one (randomly chosen) of the two resulting cells is the *daughter*, and the second one remains being the marked cell that we keep track of.

The random variable T_i follows a phase-type distribution, since it is the time to absorption for the finite Markov chain $\mathcal{Y}$ (1). The Laplace-Stieltjes transform of this random variable, and its different order moments, are given as follows (1, Proposition 1.2.2)

$$\begin{aligned} \phi_i(s) &= \mathbb{E}(e^{-sT_i}) = \mathbf{e}(i)(s\mathbf{I} - \mathbf{T})^{-1}\mathbf{t} \,, \\ \mathbb{E}(T_i^n) &= (-1)^n n! \mathbf{e}(i) T^{-n} \mathbf{e} \,, \end{aligned} \tag{1}$$

where $\mathbf{e}$ is a vector of ones, $\mathbf{e}(i)$ is a vector of zeros except for component i which is equal to 1, and the matrix $\mathbf{T}$ and the vector $\mathbf{t}$ are given by

$$\mathbf{T} = \begin{pmatrix} -(\sum_{i=1}^{M} \xi_{B,C_i} + \mu_B) & \xi_{B,C_1} & \xi_{B,C_2} & \cdots & \xi_{B,C_M} \\ \xi_{C_1,B} & -(\xi_{C_1,B} + \mu_{C_1}) & 0 & \cdots & 0 \\ \vdots & \vdots & \ddots & \vdots & \vdots \\ \xi_{C_M,B} & 0 & 0 & \cdots & -(\xi_{C_M,B} + \mu_{C_M}) \end{pmatrix},$$

$$\mathbf{t} = \begin{pmatrix} \mu_B \\ \mu_{C_1} \\ \vdots \\ \mu_{C_M} \end{pmatrix}.$$

This is equivalent to saying that the functions $\phi_i(s)$ satisfy the system of equations obtained by following a first-step argument

$$\begin{aligned} \phi_B(s)\left(\mu_B+\sum_{i=1}^{M}\xi_{B,C_i}+s\right) &= \sum_{i=1}^{M}\xi_{B,C_i}\phi_{C_i}(s)+\mu_B \ , \\ \phi_{C_i}(s)(\mu_{C_i}+\xi_{C_i,B}+s) &= \xi_{C_i,B}\phi_B(s)+\mu_{C_i} \ , \quad i\in\{1,\dots,M\} \ . \end{aligned}$$

For this CTMC, we do not need to compute the inverse of the matrix in Eq. (1), since the system above has the explicit solution

$$\begin{aligned} \phi_B(s) &= \left(\mu_B+\sum_{i=1}^{M}\xi_{B,C_i}+s-\sum_{i=1}^{M}\xi_{B,C_i}\xi_{C_i,B}(\mu_{C_i}+\xi_{C_i,B}+s)^{-1}\right)^{-1} \\ &\quad\times\left(\sum_{i=1}^{M}\xi_{B,C_i}(\mu_{C_i}+\xi_{C_i,B}+s)^{-1}\mu_{C_i}+\mu_B\right) \ , \\ \phi_{C_i}(s) &= (\mu_{C_i}+\xi_{C_i,B}+s)^{-1}\left(\xi_{C_i,B}\phi_B(s)+\mu_{C_i}\right) \ , \quad i\in\{1,\dots,M\} \ , \end{aligned}$$

and by differentiating the equations above one can obtain the different order moments $\mathbb{E}(T_i^k)$, $k\geq 1$. For example, the mean values $m_i=\mathbb{E}(T_i)$ satisfy the system of equations

$$\begin{aligned} m_B\left(\mu_B+\sum_{i=1}^{M}\xi_{B,C_i}\right) &= \sum_{i=1}^{M}\xi_{B,C_i}m_{C_i}+1 \ , \\ m_{C_i}(\mu_{C_i}+\xi_{C_i,B}) &= \xi_{C_i,B}m_B+1 \ , \quad i\in\{1,\dots,M\} \ , \end{aligned}$$

which leads to the matrix expression in Eq. (1) for $n=1$. An explicit solution can be found for the first moments

$$\begin{aligned} m_B &= \left(\mu_B+\sum_{i=1}^{M}\xi_{B,C_i}-\sum_{i=1}^{M}\xi_{B,C_i}\xi_{C_i,B}(\mu_{C_i}+\xi_{C_i,B})^{-1}\right)^{-1}\left(\sum_{i=1}^{M}\xi_{B,C_i}(\mu_{C_i}+\xi_{C_i,B})^{-1}+1\right) \ , \\ m_{C_i} &= (\mu_{C_i}+\xi_{C_i,B})^{-1}\left(\xi_{C_i,B}m_B+1\right) \ , \quad i\in\{1,\dots,M\} \ . \end{aligned}$$

One can also analyse the number of division events carried out by this cell during its lifetime, in terms of the discrete random variable N_i, described in Section 2.2, with support in $\mathbb{N}_0=\mathbb{N}\cup\{0\}$. We note that this can be seen as a discrete counterpart of the continuous phase-type random variable T_i. We can analyse this discrete random variable by computing its probability generating function

$$\varphi_i(z) \;=\; \mathbb{E}(z^{N_i}) \;=\; \sum_{n=0}^{+\infty}\mathbb{P}(N_i=n)z^n \ , \quad i\in\{B,C_1,\dots,C_M\} \ .$$

We can apply again here a first-step argument, which leads to

$$\varphi_B(z)\left(\lambda_B+\mu_B+\sum_{i=1}^{M}\xi_{B,C_i}\right) = \sum_{i=1}^{M}\xi_{B,C_i}\varphi_{C_i}(z)+\lambda_B z\varphi_B(z)+\mu_B \ ,$$

$$\varphi_{C_i}(z)(\mu_{C_i}+\xi_{C_i,B}+\lambda_{C_i}) = \xi_{C_i,B}\varphi_B(z)+\lambda_{C_i}z\varphi_{C_i}(z)+\mu_{C_i}\ , \quad i\in\{1,\ldots,M\}\ .$$

The previous system of equations can be solved as follows

$$\begin{aligned}\varphi_B(z) &= \left(\lambda_B(1-z)+\mu_B+\sum_{i=1}^{M}\xi_{B,C_i}-\sum_{i=1}^{M}\xi_{B,C_i}\xi_{C_i,B}(\xi_{C_i,B}+\mu_{C_i}+\lambda_{C_i}(1-z))^{-1}\right)^{-1}\\ &\quad\times\left(\sum_{i=1}^{M}\xi_{B,C_i}\mu_{C_i}(\xi_{C_i,B}+\mu_{C_i}+\lambda_{C_i}(1-z))^{-1}+\mu_B\right)\ ,\\ \varphi_{C_i}(z) &= (\mu_{C_i}+\xi_{C_i,B}+\lambda_{C_i}(1-z))^{-1}\left(\xi_{C_i,B}\varphi_B(z)+\mu_{C_i}\right)\ , \quad i\in\{1,\ldots,M\}\ .\end{aligned}$$

One can check that $\varphi_B(1)=\varphi_{C_1}(1)=\cdots=\varphi_{C_M}(1)=1$ as it should be. We note that in the particular case where $\lambda=\lambda_B=\lambda_{C_1}=\cdots=\lambda_{C_M}$ and $\mu=\mu_B=\mu_{C_1}=\cdots=\mu_{C_M}$, the initial position of the cell does not affect the number of division events that it will carry out (since division and death occur with the same rates in all spatial compartment). Then, by setting $\varphi_B(z)=\varphi_{C_1}(z)=\cdots=\varphi_{C_M}(z)=\varphi(z)$ we get

$$\begin{aligned}\varphi(z) &= (\mu_{C_1}+\xi_{C_1,B}+\lambda_{C_1}(1-z))^{-1}\left(\xi_{C_1,B}\varphi(z)+\mu_{C_1}\right)\\ &\Rightarrow\ \varphi(z) = \frac{\mu}{\mu+\lambda(1-z)} = \frac{\mu}{\mu+\lambda}\sum_{n=0}^{+\infty}\left(\frac{\lambda}{\mu+\lambda}\right)^n z^n\ ,\end{aligned}$$

so that $N_B=N_{C_1}=\cdots=N_{C_M}$ are just the same geometric random variable. This is to be expected since in this case these random variables represent the number of events of one type (division) until an event of a second type (death) occurs.

One can also compute the different order moments of the random variables N_i in a similar way. For example, the mean values $\hat{m}_i=\mathbb{E}(N_i)$ satisfy the system of equations

$$\begin{aligned}\hat{m}_B\left(\mu_B+\sum_{i=1}^{M}\xi_{B,C_i}\right) &= \sum_{i=1}^{M}\xi_{B,C_i}\hat{m}_{C_i}+\lambda_B\ ,\\ \hat{m}_{C_i}(\mu_{C_i}+\xi_{C_i,B}) &= \xi_{C_i,B}\hat{m}_B+\lambda_{C_i}\ , \quad i\in\{1,\ldots,M\}\ ,\end{aligned}$$

which has the following solution

$$\begin{aligned}\hat{m}_B &= \left(\mu_B+\sum_{i=1}^{M}\xi_{B,C_i}-\sum_{i=1}^{M}\xi_{B,C_i}\xi_{C_i,B}(\xi_{C_i,B}+\mu_{C_i})^{-1}\right)^{-1}\left(\sum_{i=1}^{M}\xi_{B,C_i}\lambda_{C_i}(\xi_{C_i,B}+\mu_{C_i})^{-1}+\lambda_B\right)\ ,\\ \hat{m}_{C_i} &= (\mu_{C_i}+\xi_{C_i,B})^{-1}\left(\xi_{C_i,B}\hat{m}_B+\lambda_{C_i}\right)\ , \quad i\in\{1,\ldots,M\}\ .\end{aligned}$$

Not only the different order moments, but the complete distribution of N_i, for $i\in\{B,C_1,\ldots,C_M\}$, can be found also by a first-step argument. In particular, if we define $\omega_i(n)=\mathbb{P}(N_i=n)$, $n\in\mathbb{N}_0$, we get

$$\begin{aligned}\omega_B(n)\left(\lambda_B+\mu_B+\sum_{i=1}^{M}\xi_{B,C_i}\right) &= \sum_{i=1}^{M}\xi_{B,C_i}\omega_{C_i}(n)+\lambda_B\omega_B(n-1)\ ,\\ \omega_{C_i}(n)(\lambda_{C_i}+\mu_{C_i}+\xi_{C_i,B}) &= \xi_{C_i,B}\omega_B(n)+\lambda_{C_i}\omega_{C_i}(n-1)\ , \quad i\in\{1,\ldots,M\}\ .\end{aligned}$$

This leads to a recursive solution where the probabilities $\{w_i(n),\ i \in \{B, C_1, \ldots, C_M\}\}$ can be obtained if one has already obtained the probabilities $\{w_i(n-1),\ i \in \{B, C_1, \ldots, C_M\}\}$. Thus, one can start by computing the probabilities $\{w_i(n=1),\ i \in \{B, C_1, \ldots, C_M\}\}$, given that we know the probabilities $\omega_i(n-1) = \omega_i(0) = \varphi_i(0)$.

1.2 Number of division events at each compartment

Here, we focus on the number of division events occurring in compartment j, for a cell initially in compartment i, in terms of the random variable $N_i(j)$ as described in Section 2.2. It is clear that $N_i = N_i(B) + N_i(C_1) + \cdots + N_i(C_M)$ for any $i \in \{B, C_1, \ldots, C_M\}$, and if we define $\varphi_i(z;j) = \mathbb{E}(z^{N_i(j)})$, by a first-step argument, for example for $j = B$, we get:

$$\begin{aligned}
\varphi_B(z;B)\left(\lambda_B + \mu_B + \sum_{i=1}^{M} \xi_{B,C_i}\right) &= \sum_{i=1}^{M} \xi_{B,C_i}\varphi_{C_i}(z;B) + \lambda_B z \varphi_B(z;B) + \mu_B \ , \\
\varphi_{C_i}(z;B)(\mu_{C_i} + \xi_{C_i,B}) &= \xi_{C_i,B}\varphi_B(z;B) + \mu_{C_i} \ , \quad i \in \{1, \ldots, M\} \ ,
\end{aligned}$$

which leads to the solutions

$$\begin{aligned}
\varphi_B(z;B) &= \left(\lambda_B(1-z) + \mu_B + \sum_{i=1}^{M} \xi_{B,C_i} - \sum_{i=1}^{M} \xi_{B,C_i}(\mu_{C_i} + \xi_{C_i,B})^{-1}\xi_{C_i,B}\right)^{-1} \\
&\quad \times \left(\sum_{i=1}^{M} \xi_{B,C_i}(\mu_{C_i} + \xi_{C_i,B})^{-1}\mu_{C_i} + \mu_B\right) , \\
\varphi_{C_i}(z;B) &= (\mu_{C_i} + \xi_{C_i,B})^{-1}(\xi_{C_i,B}\varphi_B(z;B) + \mu_{C_i}) \ , \quad i \in \{1, \ldots, M\} \ .
\end{aligned}$$

Similar equations can be found for $j \in \{C_1, \ldots, C_M\}$. We can also compute the different order moments of these random variables. For example, if we define $m_i(j) = \mathbb{E}(N_i(j))$, we can get the equations

$$\begin{aligned}
m_B(B)\left(\mu_B + \sum_{i=1}^{M} \xi_{B,C_i}\right) &= \sum_{i=1}^{M} \xi_{B,C_i} m_{C_i}(B) + \lambda_B \ , \\
m_{C_i}(B)(\mu_{C_i} + \xi_{C_i,B}) &= \xi_{C_i,B} m_B(B) \ , \quad i \in \{1, \ldots, M\} \ ,
\end{aligned}$$

with general solution

$$\begin{aligned}
m_B(B) &= \left(\mu_B + \sum_{i=1}^{M} \xi_{B,C_i} - \sum_{i=1}^{M} \xi_{B,C_i}(\mu_{C_i} + \xi_{C_i,B})^{-1}\xi_{C_i,B}\right)^{-1} \lambda_B \ , \\
m_{C_i}(B) &= (\mu_{C_i} + \xi_{C_i,B})^{-1}\xi_{C_i,B} m_B(B) \ , \quad i \in \{1, \ldots, M\} \ .
\end{aligned}$$

Similar arguments apply for computing the complete probability mass function of $N_i(j)$. In particular, if we define $\alpha_i(j;n) = \mathbb{P}(N_i(j) = n)$, we can get the recursive relationship

$$\alpha_B(B;n)\left(\lambda_B + \mu_B + \sum_{i=1}^{M} \xi_{B,C_i}\right) = \sum_{i=1}^{M} \xi_{B,C_i}\alpha_{C_i}(B;n) + \lambda_B \alpha_B(B;n-1) \ ,$$

$$\alpha_{C_i}(B;n)(\mu_{C_i}+\xi_{C_i,B}+\lambda_{C_i}) \;=\; \xi_{C_i,B}\alpha_B(B;n)+\lambda_{C_i}\alpha_{C_i}(B;n)\ , \quad i\in\{1,\ldots,M\}\ ,$$

which can be solved recursively starting at $n=1$, for which $\alpha_B(B;n-1)=\alpha_B(B;0)=\varphi_B(0;B)$, as has been previously computed.

1.3 Identifying the compartment before death

Cell death probabilities $\beta_i(j)$ as decribed in Section 2.2, for $i,j\in\{B,C_1,\ldots,C_M\}$, can be computed in terms of the solution of systems of linear equations. For example, if we set $j=B$, one gets

$$\begin{aligned}\beta_B(B)\left(\mu_B+\sum_{i=1}^{M}\xi_{B,C_i}\right) &= \sum_{i=1}^{M}\xi_{B,C_i}\beta_{C_i}(B)+\mu_B\ ,\\ \beta_{C_i}(B)(\mu_{C_i}+\xi_{C_i,B}) &= \xi_{C_i,B}\beta_B(B)\ , \quad i\in\{1,\ldots,M\}\ ,\end{aligned}$$

with general solution

$$\begin{aligned}\beta_B(B) &= \left(\mu_B+\sum_{i=1}^{M}\xi_{B,C_i}-\sum_{i=1}^{M}\xi_{B,C_i}(\mu_{C_i}+\xi_{C_i,B})^{-1}\xi_{C_i,B}\right)^{-1}\mu_B\ ,\\ \beta_{C_i}(B) &= (\mu_{C_i}+\xi_{C_i,B})^{-1}\xi_{C_i,B}\beta_B(B)\ , \quad i\in\{1,\ldots,M\}\ ,\end{aligned}$$

and similar solutions can be found for $j\in\{C_1,\ldots,C_M\}$.

1.4 Number of cells within a given cell's genealogy

When we analyse the number of division events in the previous sections, a single cell is tracked until its death, and the rest of the cell population is not studied. We can propose here to keep track of the entire population resulting from the original cell, and to count the total number of cells that appear within the original cell's genealogy. Thus, the discrete random variable G_i defined in Section 2.2 represents a measure of the replication potential of this single cell in the system. For example, $G_B=11$ for the stochastic trajectory shown in **Figure 2**, representing the genealogy of a cell starting in the blood.

We focus in this section on computing $\mathbb{E}(G_i)$. We first note that for some parameters values, one might have $\mathbb{E}(G_i)=+\infty$ (in the situation in which the cell's genealogy does not go to extinction). If we define $\tilde{m}_i=\mathbb{E}(G_i)$, these quantities need to satisfy, by a first-step argument

$$\begin{aligned}\tilde{m}_B\left(\mu_B+\sum_{i=1}^{M}\xi_{B,C_i}+\lambda_B\right) &= \sum_{i=1}^{M}\xi_{B,C_i}\tilde{m}_{C_i}+\lambda_B(2+2\tilde{m}_B)\ ,\\ \tilde{m}_{C_i}(\mu_{C_i}+\xi_{C_i,B}+\lambda_{C_i}) &= \xi_{C_i,B}\tilde{m}_B+\lambda_{C_i}(2+2\tilde{m}_{C_i})\ , \quad i\in\{1,\ldots,M\}\ .\end{aligned}$$

Since it is clear that one should get $\tilde{m}_i\geq 0$, we find the (finite and non-negative) solution

$$\begin{aligned}\tilde{m}_B &= \left(\mu_B+\sum_{i=1}^{N}\xi_{B,C_i}-\lambda_B-\sum_{i=1}^{N}\xi_{B,C_i}(\mu_{C_i}+\xi_{C_i,B}-\lambda_{C_i})^{-1}\xi_{C_i,B}\right)^{-1}\\ &\quad\times\left(\sum_{i=1}^{N}\xi_{B,C_i}(\mu_{C_i}+\xi_{C_i,B}-\lambda_{C_i})^{-1}2\lambda_{C_i}+2\lambda_B\right)\ ,\end{aligned}$$

$$\tilde{m}_{C_i} \quad = \quad (\mu_{C_i} + \xi_{C_i,B} - \lambda_{C_i})^{-1}(\xi_{C_i,B}\tilde{m}_B + 2\lambda_{C_i}) \ , \quad i \in \{1, \dots, N\} \ ,$$

under parametric regimes satisfying Eq. (1) and Eq. (2) in the main manuscript.

2 APPROXIMATION FOR THE NUMBER OF CELLS IN THE GENEALOGY

As we mentioned in Section 2.3, the number of cells in the genealogy of a single cell in the original process can be approximated by the number of cells in the genealogy of the corresponding branching process with division rate $\bar{\lambda}$ and death rate $\bar{\mu}$. In this section, we focus on how to compute this mean number of cells under this approximation.

Let us consider the branching process with rates $\bar{\mu}$ and $\bar{\lambda}$. Let us count the number of cells in the entire family tree, starting from one cell. For this, time is not relevant so we can count cells by generations. Let us introduce

$$Y_n \quad = \quad \text{“\textit{number of cells in generation} } n\text{”},$$

with $Y_1 = 1$, and let us define

$$S_n = \sum_{m=1}^{n} Y_m \ .$$

It is clear that the mean total family size is $S = \lim_{n\to+\infty} S_n$. Let

$$p_0 = \frac{\bar{\mu}}{\bar{\mu}+\bar{\lambda}} \ , \qquad p_2 = \frac{\bar{\lambda}}{\bar{\mu}+\bar{\lambda}} \ , \qquad \text{and} \qquad \phi(z) = p_0 + p_2 z^2 \ ,$$

where the family dies out (and $\mathbb{E}(S)$ is finite) if and only if $p_2 < 1/2$. We notice that

$$\mathbb{E}(S) = p_0 + p_2(1 + 2\mathbb{E}(S)) \ ,$$

so that

$$\mathbb{E}(S) = \frac{1}{1-2p_2} = \frac{\bar{\mu}+\bar{\lambda}}{\bar{\mu}-\bar{\lambda}} \ .$$

Instead of computing the mean value $\mathbb{E}(S)$, one can compute the probability generating function of S. To this end, we first find a relationship between S_{n+1} and S_n, using the probability generating functions, as follows

$$h_n^{(k)}(z) = \mathbb{E}(z^{S_n}|Y_1 = k) = \sum_{k=1}^{+\infty} \mathbb{P}(S_n = k|Y_1 = k) z^k \ .$$

Now we notice that

$$h_n^{(k)}(z) = (h_n^{(1)}(z))^k \ ,$$

because the trees founded by each of the k initial cells are independent. Next, we write

$$h_{n+1}^{(1)}(z) \ = p_0 z + p_2 z h_n^{(2)}(z) \ = p_0 z + p_2 z (h_n^{(1)}(z))^2 \ = z\phi(h_n^{(1)}(z)) \ .$$

The probability generating function of the number of cells in the tree,

$$h(z) = \lim_{n \to +\infty} h_n^{(1)}(z) \; ,$$

satisfies

$$h(z) = z\phi(h(z)) \; .$$

That is, we can write

$$\begin{aligned} h &= zp_0 + zp_2h^2 \; , \\ 0 &= zp_0 - h + zp_2h^2 \; , \\ h(z) &= \frac{-1 \pm \sqrt{1 - 4z^2p_0p_2}}{2zp_2} \; , \end{aligned}$$

and the complete probability mass function of S, as well as its different order moments, can be obtained by successive derivation of the expression above.

REFERENCES

1 .He QM. *Fundamentals of Matrix-Analytic Methods.* Springer, New York (2014).
